# *miR-625-3p* regulates oxaliplatin resistance by targeting MAP2K6-p38 signalling in human colorectal adenocarcinoma cells

**DOI:** 10.1038/ncomms12436

**Published:** 2016-08-16

**Authors:** Mads Heilskov Rasmussen, Iben Lyskjær, Rosa Rakownikow Jersie-Christensen, Line Schmidt Tarpgaard, Bjarke Primdal-Bengtson, Morten Muhlig Nielsen, Jakob Skou Pedersen, Tine Plato Hansen, Flemming Hansen, Jesper Velgaard Olsen, Per Pfeiffer, Torben Falck Ørntoft, Claus Lindbjerg Andersen

**Affiliations:** 1 Department of Molecular Medicine, Department of Clinical Medicine, Aarhus University Hospital, Palle Juul-Jensens Boulevard 99, DK-8200 Aarhus, Denmark; 2 Department of Proteomics, Faculty of Health and Medical Sciences, Novo Nordisk Foundation Center for Protein Research, University of Copenhagen, Blegdamsvej 3b, DK-2200 Copenhagen, Denmark; 3 Institute of Clinical Research, University of Southern Denmark, Winsløwparken 19, DK-5000 Odense, Denmark; 4 Department of Pathology, Institute of Clinical Research, Odense University Hospital, Winsløwparken 15, DK-5000 Odense, Denmark; 5 Department of Oncology, Aarhus University Hospital, Nørrebrogade 44, DK-8200 Aarhus, Denmark; 6 Department of Oncology, Odense University Hospital, Sdr. Boulevard 29, DK-5000 Odense, Denmark

## Abstract

Oxaliplatin resistance in colorectal cancers (CRC) is a major medical problem, and predictive markers are urgently needed. Recently, *miR-625-3p* was reported as a promising predictive marker. Herein, we show that *miR-625-3p* functionally induces oxaliplatin resistance in CRC cells, and identify the signalling networks affected by *miR-625-3p*. We show that the p38 MAPK activator MAP2K6 is a direct target of *miR-625-3p*, and, accordingly, is downregulated in non-responder patients of oxaliplatin therapy. *miR-625-3p*-mediated resistance is reversed by anti-miR-625-3p treatment and ectopic expression of a *miR-625-3p* insensitive MAP2K6 variant. In addition, reduction of p38 signalling by using siRNAs, chemical inhibitors or expression of a dominant-negative MAP2K6 protein induces resistance to oxaliplatin. Transcriptome, proteome and phosphoproteome profiles confirm inactivation of MAP2K6-p38 signalling as one likely mechanism of oxaliplatin resistance. Our study shows that *miR-625-3p* induces oxaliplatin resistance by abrogating MAP2K6-p38-regulated apoptosis and cell cycle control networks, and corroborates the predictive power of *miR-625-3p.*

## Abstract (web summary)

Oxaliplatin resistance in colorectal cancers is a major clinical problem, and predictive markers are urgently needed. Here, the authors show that *miR-625-3p* expression reduces the sensitivity of colorectal cancer cells to oxaliplatin by targeting the kinase MAP2K6, an activator of the MAPK14 pathway.

## Graphical abstract

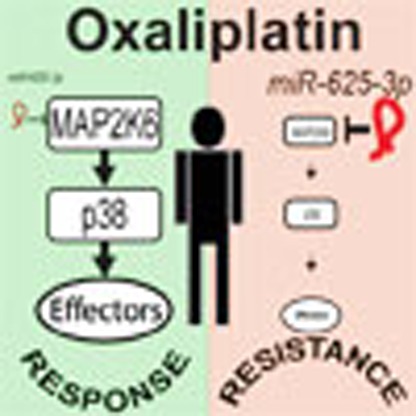

Colorectal cancer (CRC) is the third most commonly diagnosed malignant disease in the western world^1^. Approximately 25% of the patients present with a disseminated, stage IV disease and in further 10–15% of patients with initially localized disease, metastases will develop within 5 years. However, no predictive biomarker for standard chemotherapeutic treatment is available and as many as 50% of the patients do not obtain an objective response to first-line treatment^2^. Thus, the identification of predictive biomarkers for response is of great importance.

MicroRNAs (miRNAs) are endogenous, small non-coding RNAs that play essential roles in the regulation of gene expression^3^, and which have been linked to chemotherapy resistance^4^. Recently, *miR-625-3p* was reported to be positively associated with lack of response to first-line oxaliplatin (oxPt)-based treatment in two independent cohorts of patients with metastatic CRC (mCRC)^5^. While that study suggested high expression of *miR-625-3p* to be a novel predictive marker for oxPt-resistance in a subset of mCRC patients, a possible functional relationship between *miR-625-3p* and cellular drug sensitivity was not examined.

Here, we have constructed a transposon-based doxycycline (DOX) inducible vector to investigate the role of *miR-625-3p* in modulating oxPt sensitivity in CRC cells *in vitro*. Our results show that on exposure to oxPt ectopic expression of *miR-625-3p* increases cell viability by decreasing apoptosis. Furthermore, we have identified direct and indirect targets of *miR-625-3p* dysregulation in these cells and in mCRC patients treated with first-line oxPt. We show that *miR-625-3p* directly targets and inhibits the mitogen activated protein kinase (MAPK) kinase MAP2K6 (also known as MKK6). As a consequence, we find that *miR-625-3p*-induced resistance is associated with reduced MAP kinase signal transduction after genotoxic stress leading to a reduction of p38-mediated apoptosis and an increase in cell cycle progression signals.

## Results

### Ectopic expression of *miR-625-3p* promotes oxPt resistance

We constructed a *Sleeping Beauty* (SB) transposon vector (pSBInducer), which allows for stable expression of small interfering RNAs (siRNAs) and miRNAs in a DOX-inducible manner (Supplementary Fig. 1), and consequently, robust downregulation of targeted genes in mammalian cells (Supplementary Fig. 2).

**Figure 1:**
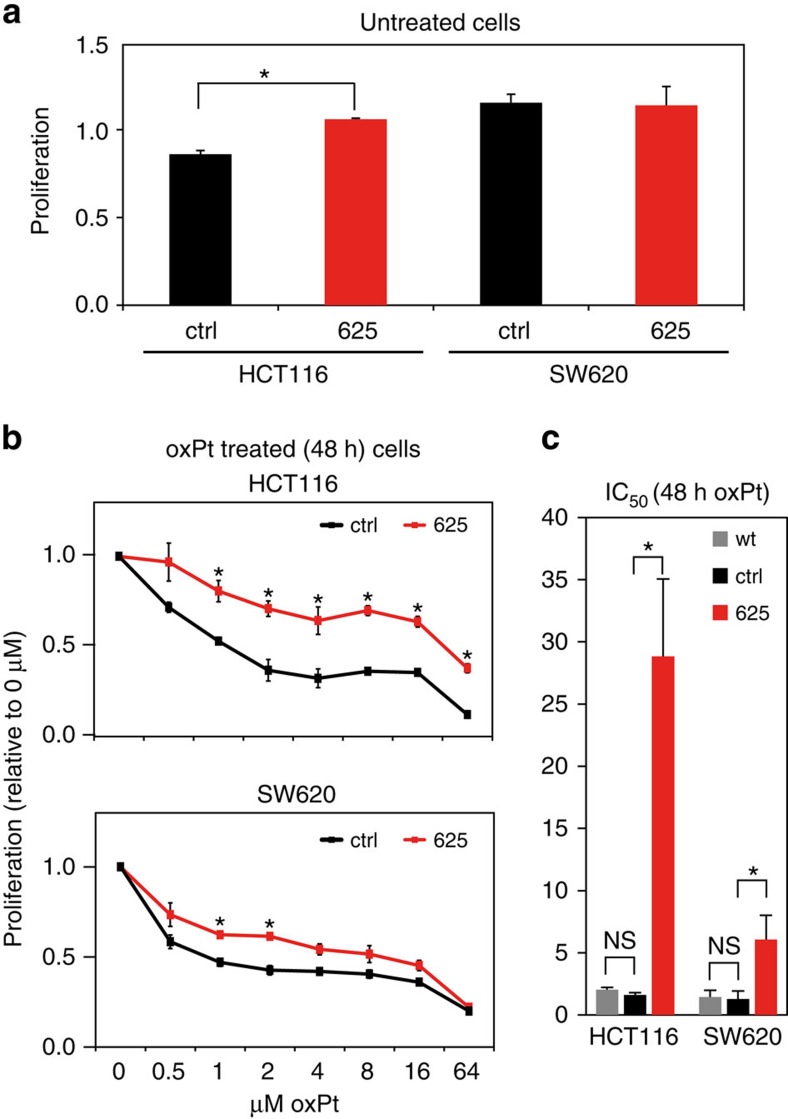
Ectopic expression of *miR-625-3p* is associated with increased viability in oxPt medium. (**a**) Cell proliferation upon DOX induction of *miR-625-3p* in the CRC cell lines HCT116.625, SW620.625 and control cells expressing a scrambled shRNA was determined by an MTT assay after 72 h of growth. Displayed as mean±s.e.m. (*n*=3). (**b**) Cell proliferation after 48 h of oxPt treatment was assessed by MTT in DOX-induced HCT116.625, SW620.625. Displayed relative to untreated cells as mean±s.e.m. (*n*=3). (**c**) IC_50_ values were calculated on the basis of experiments from **b** as well as from wild-type cells not subjected to pSBInducer transposition. Displayed as mean IC_50_±s.e.m. (*n*=3). *P≤0.05 (*t*-test); NS, not significant.

We used pSBInducer to introduce *miR-625-3p* expression (or control shRNA designed not to target any human transcripts) in the microsatellite stable and microsatellite instable CRC cell lines SW620 and HCT116, respectively (Supplementary Fig. 1). Forty-eight hours of DOX induction raised the level of *miR-625-3p* approximately three-fold in HCT116.625 cells, which is comparable to the previously reported difference in *miR-625-3p* expression between responder and non-responder patients (Supplementary Fig. 3)^5^. In SW620.625 cells, DOX treatment induced *miR-625-3p* by more than 400 fold (Supplementary Fig. 3). Ectopic expression of *miR-625-3p* had no significant effect on cell growth in SW620 cells, whereas in HCT116 cells, a slight (28%) increased viability was observed (Fig. 1a).

DOX-induced SW620.625, HCT116.625 and control cells were next treated with increasing concentrations of oxPt for 48 h and cell viability assessed. In both cell lines *miR-625-3p* induction increased oxPt resistance over a range of concentrations (Fig. 1b), which translated into an increase in the half maximum inhibitory concentration IC_50_ (causing 50% inhibition of viability) from 1.6 μM in HCT116.ctrl to 28.8 μM in HCT116.625, and from 1.3 μM in SW620.ctrl control cells to 6.1 μM in SW620.625 cells (Fig. 1c). There was no difference in IC_50_ between vector control cells and their parental wild-type counterparts (Fig. 1c). This indicates that *miR-625-3p* functionally is associated with increased resistance to oxPt in CRC cells.

### Increased *miR-625-3p* expression reduces oxPt-induced cell death

**Figure 2:**
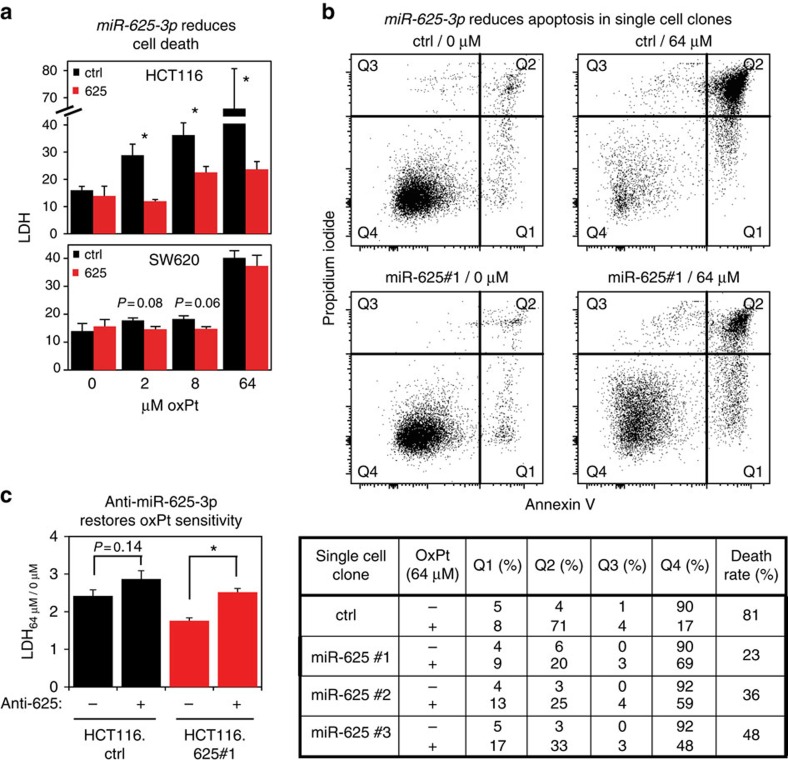
*miR-625-3p* inhibits oxPt-induced cell death in CRC cell lines. (**a**) DOX-induced HCT116.625 and SW620.625 together with control cells were treated for 48 h with oxPt. Cell death was determined with the LDH assay as 100%*(LDH_medium_/(LDH_medium_+LDH_lysate_)). Displayed as mean±s.e.m. (*n*=3). (**b**) DOX-induced HCT116.625 and HCT116.ctrl single colony-derived cells were treated with media containing 0 or 64 μM oxPt for 48 h. In the top panel, representative results from HCT116.ctrl and HCT116.625#1 single cell clones are shown. Quadrants Q1, Q2 and Q3 contain early apoptotic, late apoptotic and necrotic cells, respectively, while quadrant Q4 contains living cells. The bottom panel reports the fraction of cells in each quadrant for three independent HCT116.625 single cell clones. The death rate was calculated as 100%*(1−[Q4_64 μM_/Q4_0 μM_]). (**c**) HCT116.625#1 and HCT116.ctrl cells were induced with DOX and transfected with 20 nM anti-miR-625-3p oligo. Twenty-four hours after transfection, cells were cultivated in 0 or 64 μM oxPt for 48 h before cell death was assessed by LDH assay. Data are presented as mean increase in 64 μM oxPt-induced cell death±s.e.m. (*n*=5). *P≤0.05 (*t*-test).

To determine whether inhibition of cell death was a contributing factor to the observed oxPt resistance in HCT116.625 and SW620.625 cells, we performed a lactate dehydrogenase activity (LDH) assay. Induction of *miR-625-3p* in HCT116.625 cells inhibited drug-induced cell death when exposed to oxPt (Fig. 2a). A small decrease in cell death was also observed for 2 and 8 μM oxPt in *miR-625-3p* overexpressing SW620.625 cells although this was only borderline significant (Fig. 2a).

To confirm that the oxPt resistance phenotype was a general consequence of *miR-625-3p* induction, we used a flow cytometry-based Annexin-V/propidium iodide (PI) cell death assay on three randomly selected, independent HCT116.625 single cell clones (these are biological replicates since *Sleeping Beauty* mediated transposition is near-random and individual low-passage cell clones harbour unique pSBInducer integrations^6^). In agreement with the LDH assay, the Annexin-V/PI assay demonstrated that *miR-625-3p* indeed reduced oxPt-induced cell death (Fig. 2b). The percentage of apoptotic cells in non-treated cells was similar in control and *miR-625-3p* cell clones, while the death rate upon exposure to oxPt was reduced from 81% in control cells to below 50% in the HCT116.625 cell clones. The same experiment was also performed with a single cell-derived SW620 clone, which revealed a similar effect (reduction in death rate from 51% in SW620.ctrl to 33% in SW620.625 cells; Supplementary Table 1).

To investigate whether sensitivity towards oxPt could be restored by reducing *miR-625-3p* levels, the most oxPt-resistant HCT116.625 clone (clone #1) was transfected with an inhibitor of *miR-625-3p* (an anti-miR). The anti-miR significantly increased oxPt sensitivity towards 64 μM oxPt as assessed by LDH assay compared with mock transfected HCT116.625#1 cells (Fig. 2c). Anti-miR treatment also increased the sensitivity of control cells toward oxPt, although the difference was only borderline significant (*P*=0.140, *t*-test), presumably reflecting an effect of downregulating the endogenous *miR-625-3p* (Fig. 2c). Finally, decreased apoptosis in the HCT116.625 single cell clones upon exposure to oxPt was also supported by xCELLigence real-time proliferation assays (Supplementary Fig. 4).

In conclusion, our data demonstrate that ectopic expression of *miR-625-3p* promotes resistance towards oxPt in CRC cells, and that this resistance is caused, at least in part, by inhibition of oxPt-induced cell death.

### *miR-625-3p* transcripts are associated with oxPt response

**Figure 3:**
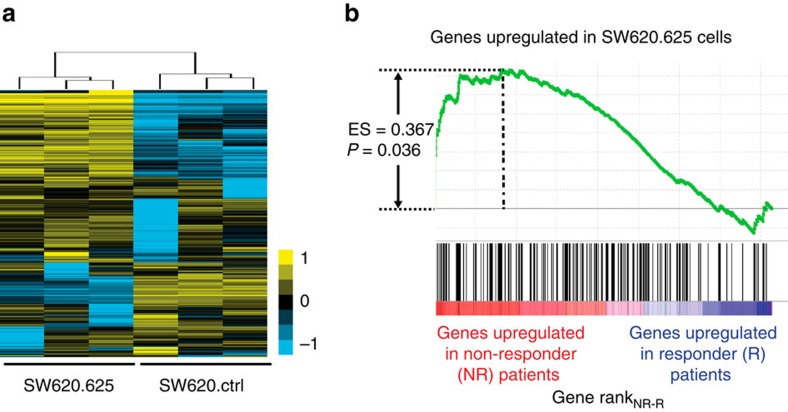
*miR-625-3p* regulates networks associated with therapy response. (**a**) Unsupervised clustering of the most variable probe sets across three SW620.625 and three SW620.ctrl samples after DOX induction. (**b**) Expression profiles of primary tumours from first-line oxPt-treated mCRC patients were generated and 20,693 genes ranked according to difference in median expression between non-responder (*n*=9) and responder (*n*=17) patients. Genes upregulated in the SW620.625 cells (black vertical bars) were significantly associated with the non-responder phenotype (enrichment score=0.367, *P*=0.036, Kolmogorov–Smirnov test).

To identify genes associated with the oxPt-resistant phenotype, transcriptional profiles of DOX-induced SW620.625 and SW620.ctrl cells were generated (Fig. 3a). We reasoned that a stronger impact on target mRNAs would be seen in SW620.625 cells as compared with HCT116.625 cells owing to the higher *miR-625-3p* levels in the former (Supplementary Fig. 3).

In total, 216 and 163 genes were up- and downregulated, respectively, in *miR-625-3p* expressing SW620.625 cells (absolute fold change >1.5; Supplementary Data 1). We noted upregulation of several genes encoding ATP-binding cassette (ABC) transporter proteins (for example, *ABCA6*, FC=17.4; and *ABCA9*, FC=2.8, see Supplementary Data 1), however, the particular ABC proteins previously implicated in multi-drug resistance (for example, *MDR1/ABCB1* and *MRP1/ABCC1*) were not dysregulated. Since no obvious pathways or single genes with strong connection to drug resistance were identified, we addressed whether the dysregulated genes might be relevant in a clinical setting. To this end, we profiled 26 microsatellite stable primary tumours from mCRC patients receiving oxPt-based therapy as first-line treatment. Objective best response to treatment was then used as phenotype labels (Non-responder and Responder) in a gene set enrichment analysis^7^. Interestingly, we found enrichment for SW620.625 upregulated genes among the non-responding patients (Fig. 3b). These data indicate a clinical relevance for the oxPt-resistant phenotype induced by ectopic *miR-625-3p* overexpression.

### The MAPK kinase MAP2K6 is a direct target of *miR-625-3p*

**Figure 4:**
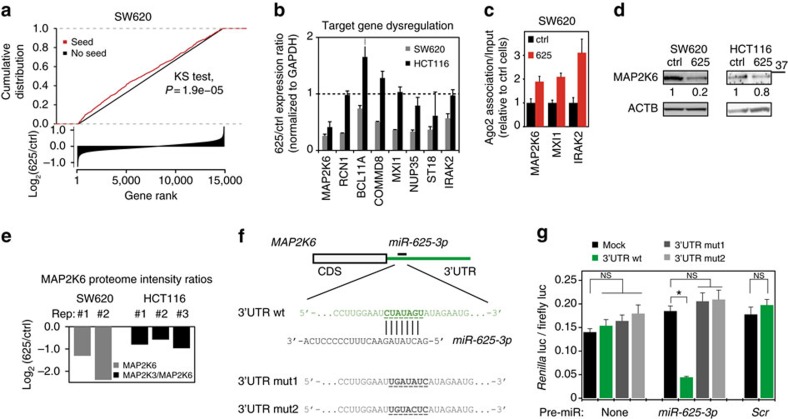
MAP2K6 is a direct and functional target of *miR-625-3p.* (**a**) Genes were ranked according to differential expression after *miR-625-3p* induction in SW620 cells (log_2_(625/ctrl) (bottom panel). The cumulative distribution of genes with a *miR-625-3p* seed sequence (red) was significantly different from the distribution of genes without a seed sequence (black) (*P*=1.9*10^−5^, Kolmogorov–Smirnov test; top panel). Note that for clarity, the log_2_(625/ctrl)-scale is truncated at −1 and 1. (**b**) The 625/ctrl-expression ratios of eight candidate target genes were determined by qRT–PCR after induction of *miR-625-3p* (or control) in SW620 cells and HCT116 CRC cells. Shown are mean expression ratio±s.e.m. (*n*=3). (**c**) qRT–PCR quantification of candidate target genes in RNA from AGO2-associated precipitates and normalized to *GAPDH* in input. Mean association±s.e.m. (*n*=3) displayed relative to control cells. (**d**) Representative western blots of MAP2K6 in SW620 and HCT116 cells after DOX-induction for 48 h. β-Actin (ACTB) was used as loading control. Quantification of MAP2K6 band intensities (normalized to ACTB) is indicated. (**e**) Quantification of MAP2K6 downregulation after induction of *miR-625-3p* as determined by mass-spec proteome analysis of two (SW620) or three (HCT116) independent DOX inductions. Displayed as log_2_ mean peptide intensity ratio. For SW620 data, 20–45 kDa proteins were excised from a denaturing gel and subjected to unlabelled proteome quantification. For HCT116 data, we used isotope-labelled whole cell lysates described below (see Fig. 6a). Note that while one MAP2K6 specific peptide was quantified in the SW620 lysates, only peptides (*n*=2) shared between MAP2K3 and MAP2K6 were detected in HCT116 cells. (**f**) Structure of the 3′UTR of MAP2K6 (ENSG00000108984, *miR-625-3p* binding site at 3′UTR position 173–180). The close-up depicts *miR-625-3p* annealed to the wild-type target sequence (underlined) as well as the two mutated sequences used in **g**. (**g**) Mean normalized Renilla Luc signal±s.e.m. (*n*=3) from HEK293T cells 24 h after transfection with psiCHECK-2 reporter containing MAP2K 3′UTR, either of the mutated 3′UTR sequences shown in **f** or mock. Experiments where a *miR-625-3p* or control (*Scr*) pre-miR were co-transfected together with psiCHECK-2 are indicated. *P<0.05 (*t*-test); NS, not significant.

To identify *miR-625-3p* target genes, we searched the transcriptional profile for mRNAs with *miR-625-3p* target sequences that were downregulated in the SW620.625 cells. Overall, we found enrichment for mRNAs containing the *miR-625-3p* 7-mer target sequence (CTATAGT) in their 3′UTR among downregulated genes (Fig. 4a). To select putative target genes for experimental validation, we used the miRmap tool, which applies multiple predictors to generate a combined score of miRNA–mRNA repression strength (from 0 to 100; ref. 8). We selected the eight most downregulated genes with a *miR-625-3p* target sequence and a miRmap score above 75: *MAP2K6*, *RCN1*, *BCL11A*, *COMMD8*, *MXI1*, *NUP35*, *ST18* and *IRAK2* (Supplementary Table 2), and confirmed downregulation of these genes by quantitative real-time PCR (Supplementary Fig. 5). Next, we screened for downregulation of these genes in a set of independently induced SW620.625 and HCT116.625 cell populations (Fig. 4b). Although all genes could be validated as being downregulated in SW620.625 cells compared with control cells, only *MAP2K6* was validated in HCT116.625 cells.

We used an anti-AGO2 antibody to immunoprecipitate RNA-induced silencing complex (RISC)-associated RNA^9^, which revealed increased AGO2 association for *MAP2K6*, *MXI1* and *IRAK2* in SW620.625 cells (Fig. 4c). At the protein level, however, only MAP2K6 were robustly downregulated after *miR-625-3p* induction in SW620.625 cells (Fig. 4d and Supplementary Fig. 6). In HCT116.625 cells, we also observed reduced MAP2K6 compared with ctrl cells although the effect appeared less pronounced than in SW620.625 cells (Fig. 4d). Since MAP2K6 levels in HCT116 cells approached the detection limit of western blotting (Supplementary Fig. 7), we estimated *miR-625-3p*-associated MAP2K6 reduction by mass spectrometry quantification, which showed a mean downregulation of 3.6- and 1.7-fold in SW620.625 and HCT116.625, respectively (Fig. 4e).

The *MAP2K6* 3′UTR contains a putative 8mer *miR-625-3p* seed site with a miRmap score of 85.49 (Fig. 4f). To experimentally confirm this, an ∼175 base fragment of the *MAP2K6* 3′UTR centred around this putative seed site was cloned into the 3′UTR of a *Renilla Luciferase* reporter gene construct (3′UTR WT *Luc* reporter). When transfected into HEK293T cells together with pre-miR-625-3p, *Luc* expression was reduced with 75% as compared with mock transfected cells (that is, *Luc* reporter with no MAP2K6 3′UTR) (Fig. 4g). The reduction was specifically related to *miR-625-3p* since co-transfection with a control pre-miR (*Scr*) had no effect on *Luc* (Fig. 4g). Furthermore, specific mutation of the *miR-625-3p* seed sequence (3′UTR mut1 and mut2) completely abolished *miR-625-3p*-mediated reduction of *Luc* (Fig. 4g).

Altogether, the data strongly support that MAP2K6 is a direct and functional target of *miR-625-3p*.

### MAP2K6–MAPK14 signalling mediates oxPt response

**Figure 5:**
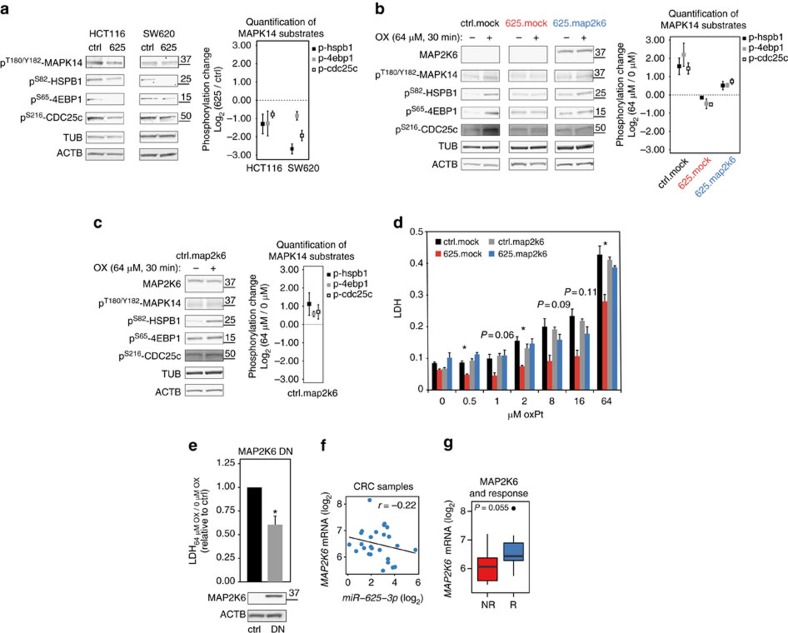
*miR-625-3p* regulates resistance to oxPt through MAP2K6 and MAPK14. (**a**) Western blotting using antibodies against the phosphorylated forms of MAPK14^T180/Y182^, HSPB1^Ser82^, 4EBP1^Ser64^ and CDC25c^S216^ in HCT116.625 and SW620.625 cells 48 h after DOX induction. β-Actin and tubulin was used as loading control (left). Quantification of HSPB1^Ser82^, 4EBP1^Ser64^ and CDC25c^S216^ western blot bands from three to five western blots normalized to α-tubulin and β-actin and displayed as log_2_(625/ctrl)±s.e.m. In one case the HSPB1^Ser82^ signal in SW620.625 was below detection level, and for this sample the median value for the two other replicates was used (right). (**b**) Changes in phosphorylation of activated p^T180/Y182^-MAPK14 and downstream substrates in HCT116.ctrl.mock, HCT116.625.mock and HCT116.625.map2k6 cells after 48 h of DOX induction followed by 30 min of 64 μM oxPt treatment (left). Quantification of HSPB1^Ser82^, 4EBP1^Ser64^ and CDC25c^S216^ substrate phosphorylation from three to five western blots normalized to α-tubulin and β-actin and displayed as oxPt-induced phosphorylation change compared with untreated cells (log_2_(64 μM/0 μM)±s.e.m. (right). (**c**) Same as **b** for the HCT116.ctrl.map2k6 cells. (**d**) Cells were DOX-induced for 48 h and treated with 0–64 μM oxPt for 48 h before cell death was determined (LDH assay). Bars represent the mean percentage of cell death±s.e.m. (*n*=3). Significant difference between HCT116.625.mock and HCT116.625.map2k cells is indicated (*P≤0.05, *t*-test). (**e**) Control HCT116 cells (ctrl) and cells expression a dominant-negative version of MAP2K6 (DN) were induced for 48 h and treated with 64 μM oxPt or left untreated for 48 h before the increase in cell death (64 μM/0 μM) was determined by LDH. Results are displayed relative to control cells (set to 1; mean±s.e.m. from *n*=4 experiments; *P≤0.05; *t*-test). Western blot against MAP2K6 (**f**) Correlation between *MAP2K6* mRNA levels and *mir-625-3p* in clinical samples (*P*=0.212, Pearson's correlation). (**g**) *MAP2K6* mRNA was downregulated in tumours from mCRC patients not responding (NR, *n*=9) compared with responders (R, *n*=17) to first-line oxPt-based therapy (*t*-test).

MAP2K6 is a dual specificity protein kinase, which transduces cellular and environmental stress signalling to its substrates, the p38 MAP kinases (MAPK11–14; ref. 10). In support of *miR-625-3p* regulating MAP2K6 signalling, we observed reduced phosphorylation of MAPK14^Tyr180/Y182^ upon *miR-625-3p-*induction (Fig. 5a). To appreciate the resulting change in MAPK14 activity, we quantified the MAPK14 substrates HSPB1^Ser82^ (ref. 11), 4EBP1^Ser65^ (ref. 12) and CDC25c^Ser216^ (ref. 13) from multiple western blots; this showed 1.7–2.5- and 1.8–6.6-fold reduction of substrate phosphorylation in HCT116.625 and SW620.625, respectively (Fig. 5a).

To mechanistically investigate the role of MAP2K6 in oxPt response in CRC cells, we stably expressed MAP2K6 lacking the *miR-625-3p* binding site in HCT116.625 cells. Western blotting confirmed high ectopic MAP2K6 levels after DOX induction compared with endogenous MAP2K6 (Fig. 5b), which is relatively lowly expressed in HCT116 cells compared with other CRC cells (Fig. 4d and Supplementary Fig. 7). First, we addressed the immediate changes in MAPK14 activity upon 30 min oxPt treatment. OxPt exposure in HCT116.ctrl.mock control cells led to increased MAPK14^Tyr180/Y182^ phosphorylation and a concurrent increase in MAPK14 activity (3.0-, 4.6- and 2.7-fold increased phosphorylation of HSPB1^Ser82^, 4EBP1^Ser65^ and CDC25c^Ser216^, respectively; Fig. 5b). However, when cells with increased *miR-625-3p* levels (HCT116.625.mock) were exposed to oxPt, we observed lack of MAPK14 activation and even a small reduction in MAPK14 substrate phosphorylation levels (Fig. 5b). In contrast, oxPt treatment of HCT116.625.map2k6 cells was associated with increased MAPK14 substrate phosphorylation (Fig. 5b), indicating that ectopic MAP2K6 was able to rescue oxPt-induced MAP2K6 signalling. Interestingly, the moderate induction of MAPK14 activity (1.4-, 1.4- and 1.7-fold increased HSPB1^Ser82^, 4EBP1^Ser65^ and CDC25c^Ser216^ phosphorylation) shows that MAP2K6 overexpression is not associated with hyperactivation of MAPK14 signalling under these conditions. To directly address whether ectopic MAP2K6 in itself made HCT116 cells hypersentitive to oxPt, we induced ectopic MAP2K6 in HCT116.ctrl cells (HCT116.ctrl.map2k6) for 48 h before treating them with oxPt for 30 min (Fig. 5c). No hyperactivation was observed, in fact the induced increase in HSPB1^Ser82^, 4EBP1^Ser65^ and CDC25c^Ser216^ phosphorylation (2.2-, 1.5- and 1.6-fold increased HSPB1^Ser82^, 4EBP1^Ser65^ and CDC25c^Ser216^ phosphorylation) was less than in HCT116.ctrl.mock cells and comparable to HCT116.625.map2k6 cells (Fig. 5c). This suggests the presence of feedback mechanisms such as the dual-specificity protein phosphatases^14^ or that other signalling components become limiting^15^.

We next investigated how ectopic expression of the *miR-625-3p* insensitive MAP2K6 variant affected the ability of *miR-625-3p* to inhibit oxPt-induced cell death (Fig. 5d). As expected, after 48 h of oxPt treatment cell death was reduced in HCT116.625.mock compared to HCT116.ctrl.mock cells. The introduction of ectopic MAP2K6, however, resensitized HCT116.625.map2k6 cells to oxPt, reaching the same level of cell death as HCT116.ctrl.mock cells. In agreement with the changes in MAPK14 activity assessed after 30 min oxPt treatment (Fig. 5b,c), the control experiment with HCT116.ctrl.map2k6 cells confirmed that expression of MAP2K6 did not lead to oxPt hypersensitivity (Fig. 5d). Taken together, these findings indicate that increased oxPt resistance mediated by *miR-625-3p* is conveyed through its target MAP2K6.

To further corroborate the importance of MAP2K6 for mediating the effect of *miR-625-3p* in CRC cells, we generated stable HCT116 cell lines expressing a dominant-negative version of MAP2K6 harbouring a K82A mutation, which abolishes kinase activity^16^. Western blotting showed the dominant-negative MAP2K6 to be expressed at many times higher level than the endogenous MAP2K6 (Fig. 5e). Dominant-negative MAP2K6 expressing cells showed a ∼40% reduction in 64 μM oxPt-induced cell death compared with control HCT116 cells (Fig. 5e), and hence, mimics the phenotype of *miR-625-3p* overexpressing cells.

We finally asked whether *MAP2K6* might be correlated with *miR-625-3p* and chemotherapy response in patients? Indeed, although not reaching significance, we found that *MAP2K6* was negatively correlated with *miR-625-3p* expression in 26 mCRC tumours (Pearson's *r*=−0.22; Fig. 5f). In addition, we also found *MAP2K6* to be slightly downregulated in non-responder patients compared with responder patients (Fig. 5g). Altogether, these data suggest that the oxPt-resistant phenotype induced by *miR-625-3p* in CRC cells operates through the direct target MAP2K6.

### *miR-625-3p* dysregulates MAPK signalling pathways

**Figure 6:**
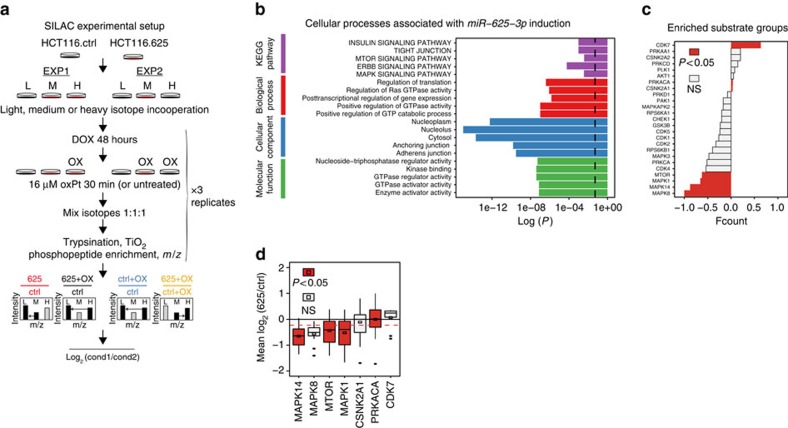
Decreased MAPK signalling is associated with *miR-625-3p* induction. (**a**) Phosphopeptide-enriched SILAC mass-spectrometry analysis was performed on HCT116.ctrl and HCT116.625 cells in two experimental setups done in parallel (‘EXP1' and ‘EXP2') each involving three experimental conditions labelled with light (‘L'), medium (‘M') or heavy (‘H') isotopes. Each experimental condition was done in triplicate. Four data sets were generated by calculating the mean log_2_ peptide intensity ratios from triplicate experiments for the following conditions: (i) HCT116.625/HCT116.ctrl (red), (ii) oxPt-treated HCT116.625/HCT116.ctrl (black), (iii) oxPt-treated HCT116.ctrl/HCT116.ctrl (blue) and (iv) oxPt-treated HCT116.625/oxPt-treated HCT116.ctrl (yellow). (**b**) Proteins with significantly dysregulated phosphopeptides after *miR-625-3p* induction were subjected to GO term and KEGG pathway enrichment analysis. The adjusted *P*-values for the most associated terms are shown. *P*=0.05 is indicated with a stippled line. (**c**) Kinase substrate enrichment analysis (KSEA) on log_2_(625/ctrl) ratios was calculated for substrate groups as the difference in the number of phosphopeptides with increased phosphorylation minus the number with decreased phosphorylation, and displayed as the fractional delta count (fcount), that is, the delta count divided with the sum of phosphopeptides in each substrate group (coloured bars indicate *P*≤0.05, hypergeometric test). (**d**) Mean log_2_(625/ctrl) phosphorylation levels were calculated for the significant substrate groups in **c**), and significance of the difference to the population mean (stippled line) calculated with a *z*-test (*P*≤0.05 indicated with coloured boxes). Mean and median are shown with a squared box and a horizontal line, respectively, the interquartile range is marked by the lower and upper hinges, respectively, and the whiskers indicate 1.5 times the interquartile range.

The results presented above indicates that the p38 MAPK subfamily (MAPK11–14) could be implicated as a mediator of platinum drug-induced stress signalling including apoptosis, a concept that has been exploited by others^17^,^18^. We therefore profiled the phosphoproteome of both untreated and oxPt-treated HCT116.ctrl and HCT116.625 cells using stable isotope labelling by amino acid (SILAC)-based mass spectrometry of TiO_2_-enriched phosphopeptides (Fig. 6a). We detected 9,423 distinct phosphopeptides on 3,217 different proteins including 177 kinases and 50 phosphatases (Supplementary Fig. 8a). We found between one and three phosphosites per protein on >75% of all detected proteins (Supplementary Fig. 8b), and detected predominantly serine phosphorylations (8,582 versus 816 and 25 threonine and tyrosine phosphopeptides, respectively; Supplementary Fig. 8c).

We first looked at the overall effect on the phosphoproteome after 48 h of increased *miR-625-3p* levels. Proteins with altered phosphorylations were mostly associated with GO terms involved with increased GTPase activity in nucleus, cytoplasm and adherence junction components, and with the mTOR, ErbB, insulin signalling and MAPK pathways (Fig. 6b). To look for changes in the activities of individual kinases, we did kinase substrate enrichment analysis (KSEA)^19^ using a merged collection of specific kinase phosphorylation site mappings obtained from the Human Protein Reference Database^20^, PhosphoSitePlus^21^ and PhosphoELM^22^ (see ‘Methods' section). KSEA indicated decreased activity of MAPK8, MAPK14, MAPK1 and MTOR kinases, and increased activity of the CDK7, PRKACA and CSNK2A1 kinases, respectively, after *miR-625-3p* induction (Fig. 6c). In agreement, the mean log_2_ ratios of MAPK14, MTOR and MAPK1 substrate groups were significantly lower than the experimental mean, and for the PRKACA substrate group it was significantly higher (Fig. 6d). Collectively, this indicates that *miR-625-3p* overexpression leads to decreased activity of MTOR, MAPK1 and the MAPK14 kinases. The latter in agreement with the notion that *miR-625-3p* targets MAP2K6.

**Figure 7:**
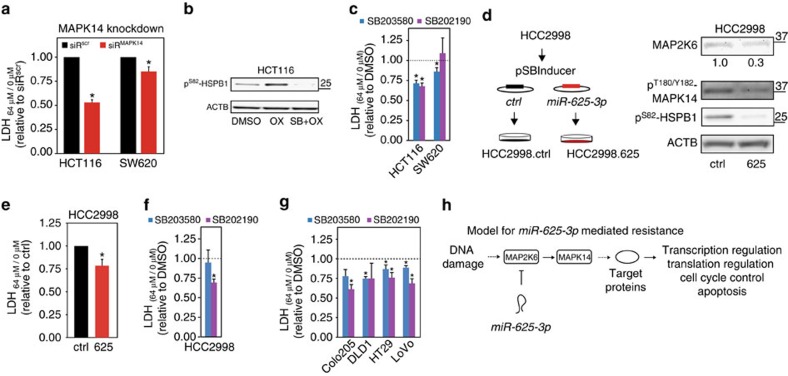
Inhibition of MAPK14 induces oxaliplatin resistance in CRC cells. (**a**) MAPK14 was specifically depleted from HCT116 and SW620 cells by transfection of a pool of MAPK14 targeting siRNAs (siR^MAPK14^) 48 h before being treated with 64 μM oxPt for 48 h (or left unexposed; see Supplementary Fig. 9 for knockdown efficiencies). The impact on cell death (64 μM/0 μM) was determined by LDH and are displayed relative to cells transfected with a scrambled siRNA (siR^scr^, set to 1). Mean±s.e.m. from at least *n*=4 experiments with ‘*' indicating a significant reduction in oxPt-induced cells death compared with siR^scr^ transfected cells (*P*≤0.05, *t*-test). (**b**) A phospho-specific western blot versus the MAPK14/MAPKAPK2 substrate Ser82-HSPB1 was applied to show increased MAPK14 activity after oxPt treatment and the inhibitory effect of 10 μM SB203580 on this activity. (**c**) HCT116 and SW620 cells were treated for 1 h with MAPK11/14 inhibitors SB203580 (10 μM, blue) or SB202190 (5 μM, purple), then exposed to 64 μM oxPt (or left unexposed) for 48 h before the increase in cell death (64 μM/0 μM) was determined by LDH. Presented relative to cells not treated with inhibitor (DMSO treated; mean±s.e.m. from at least *n*=4 experiments with ‘*' indicating a significant reduction in oxPt-induced death compared with DMSO-treated cells, *P*≤0.05, *t*-test). (**d**) Stable, inducible expression of *miR-625-3p* was generated using pSBInducer transposition in HCC2998 CRC cells (left). Phospho-specific western blot for MAP2K6 and MAPK14 activity 48 h after DOX induction of HCC2998.ctrl and HCC2998.625 cells (right). (**e**) HCC2298.ctrl and HCC2998.625 cells DOX-induced for 48 h, then treated (or left untreated) with 64 μM oxPt for 48 h before the increase in cell death (64 μM/0 μM) was determined by LDH. Results are displayed relative to control cells (set to 1; mean±s.e.m. from *n*=3 experiments; *P≤0.05, *t*-test). (**f**,**g**) HCC2998, Colo205, DLD1, HT29 and LoVo CRC cells were treated for 1 h with MAPK11/14 inhibitors SB203580 (10 μM, blue) or SB202190 (5 μM, purple) then exposed to 64 μM oxPt (or left unexposed) for 48 h before the increase in cell death (64 μM/0 μM) was determined by LDH. Displayed relative to DMSO-treated cells (mean±s.e.m. from *n*=3–4 experiments with ‘*' indicating a significant reduction, *P*≤0.05, *t*-test). (**h**) A schematic model showing how *miR-625-3p* mediated downregulation of MAP2K6 could modulate response to oxPt by abrogating MAPK14 stress-induced signalling. In the canonical model MAP2K6 in complex with MAP2K3 phosphorylates and activates MAPK14, which in turn—directly or indirectly via substrate kinases such as MAPKAPK2—activates a diverse number of target proteins central to stress-induced transcription, translation, cell cycle control and apoptosis.

To more specifically investigate the role of MAPK14 in oxPt resistance, we first inactivated MAPK14 signalling (indicated by reduced HSPB1^Ser82^ phosphorylation) in HCT116 and SW620 cells through siRNA mediated depletion of MAPK14 (Supplementary Fig. 9). MAPK14 depletion was associated with increased resistance to oxPt-induced cell death. In HCT116 cells the induced death was reduced to 50% of control cells, and in SW620 cells to 85% of controls (Fig. 7a).

Second, we used the small molecule inhibitor SB203580 to obstruct oxPt-induced MAPK14 activation in HCT116 cells as indicated by reduction of HSPB1^Ser82^ phosphorylation (Fig. 7b). SB203580 treatment reduced oxPt-induced cell death to less than 75% of control cells (Fig. 7c). A similar reduction in oxPt-induced cell death was also observed in SW620 cells (Fig. 7c). We also tested a second MAPK14 inhibitor, SB202190, which reduced the oxPt sensitivity in HCT116 cells, but not SW620 cells (Fig. 7c). Taken together, our observations in HCT116 and SW620 support that abrogation of MAPK14 signalling plays a role in oxPt resistance.

Speculating whether the observations could be generalized and extended to additional CRC cell lines we generated stable, inducible *miR-625-3p* expression in the HCC2998 CRC cell line (Fig. 7d). This line represents a tumour etiology distinct from HCT116 and SW620 by being microsatellite stable, expressing a truncated TP53 variant, and displaying a hypermutator phenotype as a consequence of a *POLE* missense mutation^23^. *miR-625-3p* levels in HCC2998.625 cells after DOX induction was increased >20-fold (Supplementary Fig. 10) and associated with decreased MAP2K6 levels as well as with decreased MAPK14 activity (Fig. 7d). Similar to HCT116 and SW620 cells, ectopic *miR-625-3p* expression reduced the 64 μM oxPt-induced cell death to ∼75% of control cells (Fig. 7e). Using the same conditions as above (Fig. 7c), chemical inhibition of MAPK14 signalling in HCC2998 cells by SB202190 also reduced oxPt induced cell death to ∼70%, while SB203580 had no effect (Fig. 7f).

To further generalize the involvement of MAPK14 signalling in oxPt response, the two MAPK14 inhibitors were applied to four additional CRC cell lines. In all four cell lines, MAPK14 inhibition reduced the sensitivity to oxPt (Fig. 5g). Taken together, these data show that inhibition of MAPK14 phenocopies the effect of *miR-625-3p* overexpression and supports the notion that the MAP2K6–MAPK14 signalling network plays a central functional role in *miR-625-3p*-induced oxPt resistance (Fig. 7h).

### The phosphoproteomic response to oxPt in CRC cells

**Figure 8:**
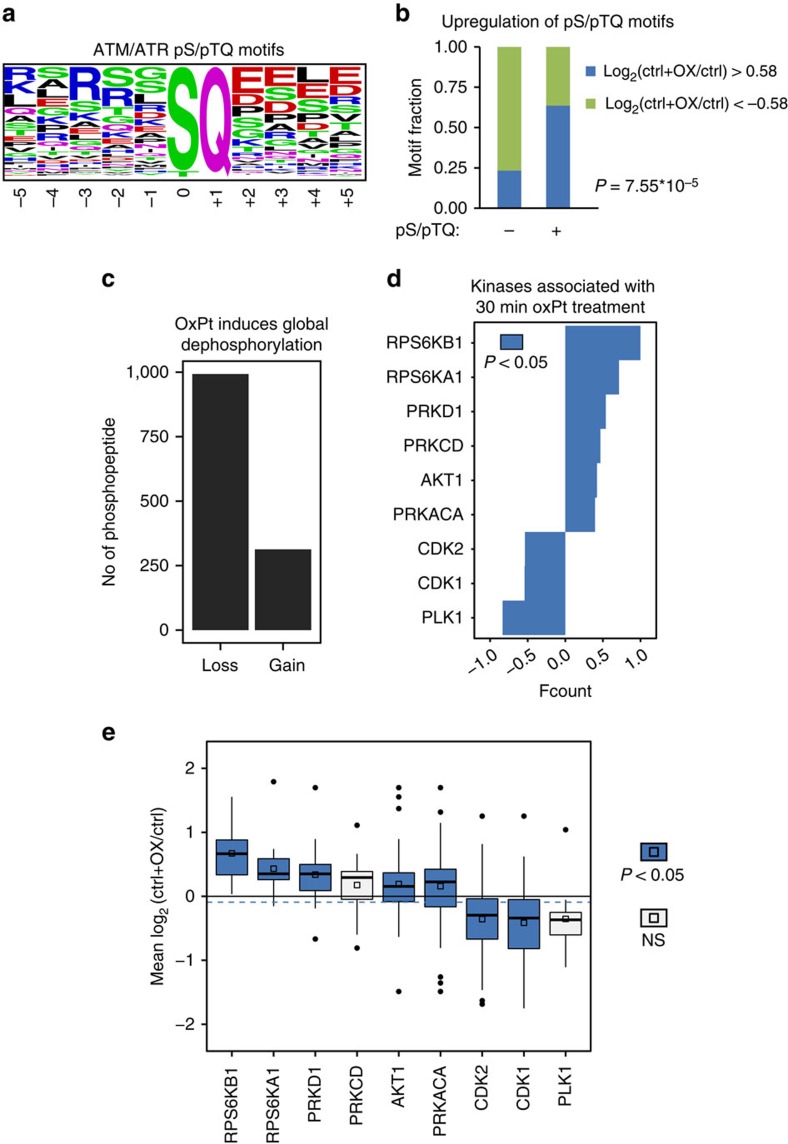
The phosphoproteome response to oxPt in CRC cells. (**a**) A sequence logo was generated based on 205 detected phosphopeptides with potential ATM/ATR phosphorylation sites (pS/pTQ). (**b**) Fisher's exact test on counts of dysregulated (log_2_(ctrl+OX/ctrl)±0.58 and false discovery rate (FDR) ≤0.1) phosphopeptides revealed significantly increased upregulation of pS/pTQ motifs after oxPt treatment. (**c**) The number of altered phosphopeptides after 30 min of 16 μM oxPt treatment were counted and grouped into peptides with decreased phosphorylation (log_2_(ctrl+OX/ctrl)<0.58) (‘Loss') and increased phosphorylation (log_2_(ctrl+OX/ctrl)>0.58) (‘Gain'). (**d**) KSEA was done on log_2_(ctrl+OX/ctrl) ratios (as described in Fig. 5). Only substrate groups with indication of altered activities after oxPt exposure are shown (*P≤0.05, hypergeometric test). (**e**) Mean log_2_ phosphorylation ratios for the nine substrate groups in **d**; (coloured boxes indicate *P*≤0.05, *z*-test). NS, not significant.

To further characterize the role of *miR-625-3p* during oxPt treatment in CRC cells, we delineated phosphorylation changes associated with the immediate (30 min) response to oxPt in control CRC cells. Totally, we detected 205 phosphopeptides with phosphoserines/threonines preceding a glutamine, which are potential substrates of ATM and ATR DNA damage response kinases (Fig. 8a)^24^. The pS/pTQ motif was enriched among peptides that had increased phosphorylation after oxPt treatment (Fig. 8b), indicating that the DNA damage response signalling was induced after 30 min of oxPt exposure. Although phosphorylation of pS/pTQ motifs increased upon oxPt treatment, the general trend was the opposite. Indeed, we found more than three times as many phosphopeptides with decreased phosphorylation (*n*=993) compared with phosphopeptides with increased phosphorylation (*n*=313) after oxPt treatment (Fig. 8c), suggesting global dephosphorylation in CRC cells immediately after oxPt exposure similar to what has been observed after cisplatin treatment^25^. Dysregulated phosphoproteins were associated with processes involved in chromatin remodelling, mitotic cell cycle, microtubule organisation and pathways such as mTOR, cell cycle, ErbB and MAPK signalling (Supplementary Fig. 11). KSEA analysis suggested increased activities of ribosomal protein S6 kinases beta-1 and alpha-1 (RPS6KB1 and RPS6KA1), and various protein kinases known to be implicated in genotoxic stress signalling (PRKACA, PRKCD and PRKD1)^26^,^27^,^28^,^29^ as well as AKT1 (Fig. 8d). Reduced activities were found for cyclin-dependent kinase 1 and 2 (CDK1 and CDK2) and polo-like kinase 1 (PLK1; Fig. 8d), in agreement with all three being positively involved in cell cycle progression and inhibition of DNA damage response^30^,^31^. The importance of these kinases in the immediate cellular response to oxPt was also supported by increased mean log_2_ phosphorylation ratios for the RPS6KB1, RPS6KA1, PRKD1, AKT1 and PRKACA substrate groups, and by decreased ratios for the CDK1 and CDK2 substrate groups (Fig. 8e).

### *miR-625-3p* blocks the normal cellular response to oxPt

**Figure 9:**
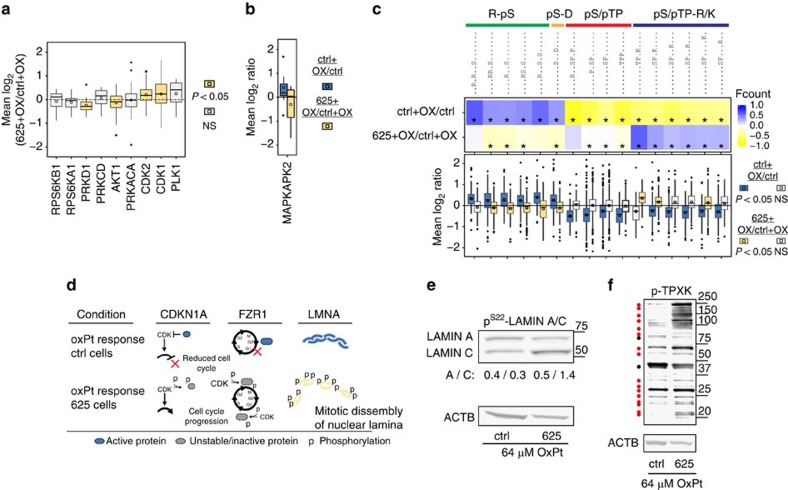
Critical components of the cellular response to oxPt are blocked in cells with increased *miR-625-3p* levels. (**a**) Mean log_2_ ratios of substrates groups involved in oxPt response were calculated for the 625+OX/ctrl+OX data. (**b**) Mean log_2_ for the MAPKAPK2 substrate group. (**c**) The most significantly altered substrate phosphorylation motifs identified for the ctrl+OX/ctrl and 625+OX/ctrl+OX experiments identified using KSEA (displayed as fcounts, *P*≤0.05 indicated with "*"). Mean log_2_ ratios for substrates with these motifs were calculated. Coloured boxes in the boxplots of **a**, **b** and **c** indicate *P*≤0.05, z-test; and NS, not significant. On the basis of similarity, the 16 individual motifs were grouped into the four motif groups indicated above. Note that the experimental mean log_2_ ratios for clarity have been omitted in **b** and **c**. (**d**) oxPt treatment in HCT116.ctrl cells led to dephosphorylation of Serine 130 (S130) of Cyclin-Dependent Kinase Inhibitor 1 (CDKN1A, also known as p21^CIP1^), which has been linked to increased stability of CDKN1A and inhibition of CDK/cyclin-mediated cell cycle progression^65^. In contrast, increased S130 phosphorylation was seen in cells with ectopic *miR-625-3p* expression. As indicated, this phosphorylation may itself be mediated by elevated CDK activity^66^. Increased CDK activity at the G1/S checkpoint or in early M phase was also indicated by S138/S151 phosphorylations on inactivated FZR1 (also known as CDH1) in *miR-625-3p* expressing cells, whereas unphosphorylated FZR1 in control cells suggested decreased CDK signalling at G0 or early G1 (ref. 67). In support of mitotic-induced nuclear lamina breakdown, increased phosphorylation was observed on multiple residues on LMNA in *miR-625-3p* cells; On the contrary, these became dephosphorylated after oxPt treatment in control cells indicating decreased cell cycle progression (also see Supplementary Fig. 14). (**e**) Western blotting against the CDK1 substrate phospho-LAMIN A/C^S22^ on lysates from oxPt-treated HCT116.ctrl and HCT116.625 cells. Quantification of bands representing Lamin A and C isoforms are indicated (normalized to β-actin signal). (**f**) Western blotting against the phosphorylated CDK motif p-TPXK on lysates from oxPt-treated HCT116.ctrl and HCT116.625 cells. Individual substrates are indicated with a dot with red and black indicating increase or decrease/no change in intensity, respectively, in HCT116.625 as compared with HCT116.ctrl cells.

We next investigated whether *miR-625-3p* expression affected the predicted activities of the oxPt-regulated kinases identified by KSEA (see Fig. 8d,e). In the 625+OX/ctrl+OX experiment, a mean log_2_ ratio different from zero is expected for kinases whose activities after oxPt treatment are altered by increased *miR-625-3p* levels, while unaffected kinases will have a mean log_2_ ratio around zero. The mean phosphorylation ratios for the oxPt-induced PRKD1 and AKT1 substrate groups were decreased, while CDK1 and CDK2 substrates on average showed increased phosphorylation levels (Fig. 9a). Strikingly, the strongest change in mean log_2_ phosphorylation ratios were found for the MAPKAPK2 substrate group (MAPK14 substrate and binding partner) whose log_2_ ratio was decreased after oxPt treatment in HCT116.625 cells (Fig. 9b). The mean log_2_ ratios for all the five substrate groups were in the opposite direction in the 625+OX/ctrl+OX as compared with the OX+ctrl/ctrl experiment. In agreement with the *miR-625-3p*-induced oxPt resistance phenotype (Fig. 2a,b), this suggested that *miR-625-3p* blocks signalling cascades central in the normal response to DNA damage.

Further, we investigated whether *miR-625-3p*-mediated blockage of oxPt-induced signalling also was evident on a phosphorylation motif level. KSEA analysis and mean log_2_ phosphorylation ratios on motif groups (that is, phosphopeptides with a similar 15 amino acid-motif centred on the phosphorylated residue) suggested that oxPt treatment of control cells led to increased kinase activities directed towards serines that are preceded by one or two basic arginine residues (R-pS motifs), or followed by an acidic aspartate (pS-D motifs) (Fig. 9c). Dephosphorylation after oxPt treatment was seen on proline directed motifs with or without a single trailing basic residue (pS/pTP-R/K and pS/pTP motifs; Fig. 9c), which are typically associated with the CDK, MAPK and GSK families^32^. In contrast, the oxPt response in the context of *miR-625-3p* led to increased pS/pTP-R/K-associated kinase activity, and generally, decreased R-pS-directed activity, while phosphorylations on pS/pTP motifs, in general, were similar in ctrl and 625 cells (Fig. 9c).

We used the network-based NetworKIN data set^33^ to identify kinases most likely associated with the differentially phosphorylated R-pS, pS-D and pS/pTP-R/K motifs (Supplementary Fig. 12). A significant association was found between the oxPt-induced motifs (R-pS and pS-D) and multiple kinase families including AKT1 and AKT2 kinases, protein kinase A, Calcium/Calmodulin-Dependent Protein Kinase II kinases (CAMKII), as well as HIPK2 and PAK kinases. The *miR-625-3p* specific pS/pTP-R/K motif was most strongly associated with cyclin-dependent kinases (CDK1, CDK2 and CDK5), and to a lesser extent with MAP kinases and TTK kinase. As expected, many of these kinases are involved in DNA damage response (for example, AKT, CAMKII, HIPK2 and PAK) and cell cycle regulation (for example, CDK, MAPK and TKK). Furthermore, several of them overlap with the kinases identified in the substrate group analysis.

To identify individual phosphoproteins associated with the observed progressive cell cycle signalling, we first defined regulatory classes based on those phosphopeptides with phosphorylation changes in opposite direction in the ctrl+OX/ctrl and 625+OX/ctrl+OX experiments (Supplementary Fig. 13). Among these, we identified several cell cycle-associated proteins, including CDKN1A, FZR1 and LAMIN A/C (Fig. 9d), with differential phosphorylation patterns that supported increased cell cycle progression in oxPt-treated HCT116.625 cells compared with control cells (Supplementary Fig. 14).

Phosphospecific western blotting against LAMIN A/C^Ser22^ (Fig. 9e)—a known CDK1 target at the onset of mitosis^34^—confirmed the observed increase in lamin phosphorylation (Supplementary Fig. 14), which is a marker of nuclear envelope disassembly during mitosis. Interestingly, increased LAMIN A/C^Ser22^ phosphorylation in oxPt-treated HCT116.625 cells appeared to be a consequence of an increase in the LAMIN C over the LAMIN A isoform (Fig. 9e).

To confirm increased CDK activity after oxPt treatment in HCT116.625 cells, we did phosphospecific western blotting against the most differentially phosphorylated CDK motif pTPXK (Fig. 9c, a target for CDKs 1 and 2, among others). This revealed increased phosphorylation in oxPt-treated HCT116.625 cells at the majority of CDK substrates consistent with increased activity (Fig. 9f).

Finally, we found that phosphorylation of ATM/ATR pT/pSQ motifs in the oxPt-treated HCT116.625 cells was significantly increased (*P*<0.05, Fisher's exact test), indicating that alteration of cell cycle signalling in these cells was not related to lack of DNA damage sensing *per se* (Supplementary Fig. 15a,b). This suggests that *miR-625-3p* acts after, or independently of, the immediate ATM/ATR-mediated DNA damage response (Supplementary Fig. 15c).

Altogether, these analyses are in support of the hypothesis that *miR-625-3p* induces blockage of signalling pathways involved in normal oxPt response, which, among other things, culminates in increased cell cycle progression signals relative to control cells.

## Discussion

Previously, we reported that high expression of *miR-625-3p* in primary tumours of mCRC patients was associated with an odds ratio above 6 for a poor response to first-line oxPt-based therapy^5^. In the present work, we have shown that *miR-625-3p* functionally leads to oxPt resistance by preventing the DNA damage response system to induce cell cycle arrest and apoptosis. Furthermore, we have identified MAP2K6 as a functional target for *miR-625-3p*, and as a mediator of *miR-625-3p*-induced oxPt resistance. To the best of our knowledge, MAP2K6 is the first functionally documented target of *miR-625-3p*, and conversely, *miR-625-3p* is the first described microRNA targeting MAP2K6. MAP2K6 (together with MAP2K3) catalyses dual phosphorylation of the TGY motif in the activation loop of the four p38 MAPK isoforms (MAPK11–14; refs 35, 36, 37), and as such conveys p38-mediated cellular stress signalling^10^. The presented results are consistent with a model were *miR-625-3p* through downregulation of MAP2K6 impairs p38-MAPK stress signalling (Fig. 7h and Supplementary Fig. 15c). It is important to emphasize, however, that our model only addresses *miR-625-3p* signalling through MAP2K6. It is likely that *miR-625-3p* additionally could mediate resistance by regulating other unknown target proteins.

On the basis of our results using chemical inhibitors and MAPK14 knockdown, and in agreement with other studies^38^,^39^, we are inclined to believe that the MAPK14 isoform of p38 is a mediator of *miR-625-3p*-induced oxPt resistance. We are aware of the discrepancy in the effect on oxPt sensitity after chemical inhibition in two (SW620 and HCC2998) out of seven cell lines tested, which we attribute to the cell-specific off-targeting effects known to exist for SB203580 and SB202190 (refs 40, 41). Our phosphoproteome data in exponentially growing unstressed CRC cells also revealed that MAPK14 was the kinase whose activity (on a substrate level) was mostly affected by *miR-625-3p* induction. Finally, oxPt treatment showed increased activity of the MAPKAPK2 kinase, which is a canonical MAPK14 substrate and binding partner responsible for nuclear translocation of MAPK14 after stress^42^. This suggests that MAPK14–MAPKAPK2 activation plays a role during oxPt response in cancer cells. Such notion is further supported by our observation of reduced activity of MAPKAPK2 in oxPt-resistant HCT116.625 cells.

We observed resistance to oxPt after *miR-625-3p* induction in all three cell models—with the strongest phenotype obtained in HCT116 cells—despite different levels of induction (3 × in HCT116, 25 × in HCC2998 and >400 × in SW620) and different degrees of MAP2K6 reduction (0.8 × in HCT116, 0.4 × in HCC2998 and 0.2 × in SW620). This indicates that the resulting level of MAP2K6 protein—rather than changes in *miR-625-3p* and MAP2K6 *per se*—determines response to oxPt. Alternative explanations include cell-specific wiring and dependencies of the MAP2K6–MAPK14 signalling pathway^15^, and diversity in a stress mediator downstream of MAPK14. An interesting candidate is TP53, which is mutated in SW620 and HCC2998 cells but wild type in HCT116. These hypotheses will have to be addressed in future studies.

Induction of p38 signalling by platinum-based drugs has been ascribed a pro-apoptotic role in multiple types of cancer cells^10^,^17^,^39^,^43^,^44^. On the other hand, p38 may also induce survival signals after cytotoxic stress^45^,^46^,^47^. In fact, MAP2K3/6-p38-MAPKAPK2/3 activation has recently emerged as a third signalling axis during DNA damage response, alongside ATM-CHEK2 and ATR-CHEK1 (refs 48, 49). In this setting, p38 signalling functions as a cell cycle checkpoint by deactivating CDC25s, cyclinE and CDK1 to prevent premature mitotic entry^48^,^50^. Thus, the outcome from dysregulated p38 signalling in drug-treated cancer cells appears to be a function of several factors including the extent and nature of the cellular insult. In that respect, we note that increased sensitivity to the topoisomerase I inhibitor irinotecan (another drug used to treat CRC patients) has been shown to correlate with decreased p38 phosphorylation in CRC patients^51^. Following this, CRC patients with high *mir-625-3p* levels and reduced MAP2K6–MAPK14 signalling, and therefore resistance to oxPt, may instead benefit from irinotecan treatment as first-line therapy.

The findings reported suggest that the expression level of *miR-625-3p*, possibly in combination with the expression level and activity of MAP2K6 and MAPK14, has the potential to serve as a biomarker for predicting response to oxPt. Since up to 20% of mCRC patients show high *miR-625-3p* expression^5^, the number of patients that potentially could benefit from quantification of the *miR-625-3p* biomarker is substantial. In addition, the observation that anti-miR-625-3p treatment makes cells with high *miR-625-3p* level responsive to oxPt, indicates that it may be possible to sensitize patients with high *miR-625-3p* expressing cancers to oxPt by *miR-625-3p* antagonist treatment before, or simultaneously with, oxPt treatment.

In conclusion, we have shown that overexpression of *miR-625-3p* in CRC cells can induce resistance to oxPt by directly targeting MAP2K6 and consequently inactivating genotoxic stress signalling conveyed by the MAP2K6–MAPK14 pathway.

## Methods

### Patients

Fresh frozen primary tumour biopsies originated from 26 patients who were treated with oxPt and 5-FU as first-line therapy for mCRC in the Departments of Odense University Hospital and Aarhus University Hospital, Denmark, as described in ref. 52. Informed consent was obtained from all the patients. The study was approved by the national ethics committees and governmental authorities in Denmark and was conducted in accordance with the Declaration of Helsinki. The patients were grouped according to objective therapy response into nine poor responders (best response being either ‘Progressive disease' or ‘Stable disease') and 17 good responders (‘Partial response' or ‘Complete response').

### Cell lines

HEK293 Flp pFRT/eGFP was a gift from Jacob Giehm Mikkelsen, Aarhus University, while CRC cells originated from the ATCC and NCI-60 repositories (kind gift from Nils Brünner, University of Copenhagen). The cell lines were authenticated by our in-house STR analysis (http://identicell.dk), and were tested negative for mycoplasma using MycoSensor PCR Assay Kit (Stratagene). All the cell lines were grown in RPMI medium 1640 with L-glutamine (Life Technologies) supplemented with 10% heat-inactivated fetal calf serum (Life Technologies). The cells were propagated in 37 °C at 90% air humidity and with 5% CO_2_. For oxPt treatment cells were first induced for 48 or 72 h with 50 ng ml^−1^ doxycycline hyclate (Sigma-Aldrich), and then cultured in medium supplemented with the indicated concentrations of oxPt (Fresenius Kabi) together with doxycycline. Chemical inhibitors SB203580 (Invivogen) and SB202190 (Invivogen) were dissolved in dimethyl sulphoxide (DMSO) and kept in aliquots at −20 °C until use. The cells were pre-incubated 1 h with inhibitor (or DMSO) supplemented medium before exposure to oxPt (or medium) containing inhibitor (or DMSO).

### Vectors

The pSBinducer vector was made by modification of the pINDUCER vector^53^. Using the pSBT-PGK-Puro plasmid as a template the SB right inverted repeat (SB-RIR) and the mouse *phosphoglycerate kinase 1* polyadenylation segment (PGApA) cloning fragments were PCR-amplified with primer pairs MreI-SB-RIR and HindIII-XcaI-c (SB-RIR), and HindIII-PacI-PGKpA and MreI-c (PGKpA), respectively (the oligos are shown in Supplementary Data 3). The SB left inverted repeat (SB-LIR) cloning fragment was amplified from pT2_CMV-eGFP-SV40-neo using primers SgrDI-SB-LIR and NheI-c (SB-LIR). The PGKpA and SB-RIR fragments were digested with *Mre*I (Fermentas) and ligated together with T4 ligase (New England Biolabs), before cloned into pUC18 using *Hin*dIII digestion. Lentiviral elements from the pINDUCER vector were removed by restriction digestion with *Sgr*DI (Fermentas) and *Nhe*I (Fermentas) and the SB-LIR fragment inserted using the same restriction sites. The PGKpA.SB-RIR fragment was excised from pUC18.PGKpA.SB-RIR using *Pac*I (Fermentas) and *Xca*I (Bst1107I, Fermentas) and introduced into pINDUCER.PGKpA.SB-LIR using the same restriction sites to generate the final pSBInducer vector. A DNA oligo constructed to allow expression of a specific shRNA when inserted into the pSBInducer vector was amplified using the universal primers miR30PCRXhoI and miR30PCREcoRI and cloned into pSBInducer using *Xho*I and *Eco*RI restriction sites (Supplementary Data 3). To generate DOX-inducible SB-mediated stable expression of recombinant FLAG-MAP2K6 and FLAG-MAP2K6DN, we first used the pINDUCER11 vector (in which the puromycin resistance gene is replaced by *EGFP*^53^) to make an EGFP expressing pSBInducer version according to the strategy outlined above. From this, we removed the tRFP-miR30-shRNA-miR30 element (see Supplementary Fig. 1) using *Age*I and *Mlu*I restriction digestion and a multiple cloning site-N-FLAG sequence (MCS-NFLAG, PCR amplified) inserted using the same restriction sites. The MAP2K6 ORF was then PCR amplified from pcMKK6wt or pcMKK6(S87A)^54^,^55^ using primers BspEI-map2k6 and NotI-map2k6 and cloned using *Bsp*EI and *Not*I to generate the pSBInducer.map2k6. Mock vector (pSBInducer.mock) was made by removing MAP2K6 by restriction cloning. In all the steps, plasmid DNA was purified with GeneElute Plasmid Miniprep Kit (Sigma-Aldrich). Correct insertion was confirmed by sequencing and with appropriate restriction digestions.

### Generation of pSBInducer cells

To generate pSBInducer cells, approximately 10 mio. cells were transfected with 1,500 ng pSBInducer.shRNA DNA (siR^EGFP^, miR-625-3p or scramble) and 1,500 ng pCMV-SB100XCO helper plasmid (or, as a negative control, 1,500 ng pUC19 DNA) using 15 μl Lipofectamine 2000 (Invitrogen) in 500 μl Opti-Mem I Medium (Gibco, Invitrogen-Life Technologies). After transfection, cells were incubated for 24 h before refreshing the media. The cells were treated with a puromycin concentration of 1 μg ml^−1^ (HCT116 and HCC2998) or 2 μg ml^−1^ puromycin (SW620 and HEK293 Flp pFRT/eGFP) for 5 days to eliminate control transfected cells. We used the tRFP fluorescence marker to sort for cell populations expressing the shRNA after induction; these cells were frozen and used for subsequent experiments. All the experiments were conducted with low-passage (<10 passages after sorting) cell populations. Single cell clones were generated from single RFP-positive cells sorted directly into 96 wells from where they were propagated and frozen. We generated MAP2K6 (or Mock) expressing cells by transposing HCT116.625 (and HCT116.ctrl) cells with pSBInducer.map2k6 (and pSBInducer.mock) as described above, except that we used FACS to isolate EGFP/tRFP double positive cells. We used western blotting and quantitative PCR with reverse transcription (qRT–PCR) to confirm expression of FLAG-MAP2K6 protein and *miR-625-3p*, respectively. Sorting was performed at the FACS Core Facility, The Faculty of Health Sciences, Aarhus University, Denmark, on a FACSAria IIII (BD Biosciences).

### Western blotting

Protein extraction and western blotting analysis were performed according to standard procedures. Antibodies were GFP (1:1,000, Abcam, ab1218), β-actin (1:25,000, Abcam, ab49900), tubulin (1:5,000, Abcam, ab7291), p38α/MAPK14 (1:500, Santa Cruz Biotechnologies, SC-81621), MAP2K6/MKK6 (1:500–1:1,000, Cell Signaling, #8550), MXI1 (1:200, Santa Cruz Biotechnologies, SC-1042), IRAK2 (1:1,000, Cell Signaling, #4367), phospho-Thr180/Tyr182-p38α/MAPK14 (1:750, Cell Signaling, #9211), phospho-Ser82-HSPB1 (1:2,000, Cell Signaling, #2406), phospho-Ser216-CDC25c (1:750, Cell Signaling, #4901), phospho-Ser65-4EBP1 (1:750–1:1,000, Cell Signaling, #9456), phospho-Ser22-Lamin A/C (1:1,000, Cell Signaling, #2026) and phospho-CDK Substrate[pTPXK] (1:1,000, Cell Signaling, #14371). Densitometrical quantification of MAP2K6 protein and MAPK14 phospho-substrates was done in ImageJ using β-actin and α-tubulin as loading controls (Supplementary Fig. 16).

### RNA extraction, reverse transcription and qRT–PCR

Total RNA from cell lines was purified using QIAzol Lysis Reagent (Qiagen) according to the manufacturer's guidelines. RNA quality and integrity was ensured according to Agilent 2100 Bioanalyzer runs (RIN score >9.5 for all samples; Agilent Technologies). Small RNA expression levels were quantified with qRT–PCR according to the protocol of the Universal cDNA synthesis kit (Exiqon) using miRCURY LNA Universal RT microRNA PCR assays (Exiqon) and SYBR Green master mix (Exiqon) according to the manufacturer's instructions. For mRNA detection, single-strand cDNA was synthesized using the Superscript Reverse Transcriptase Kit (Life Technologies) and qRT–PCR was performed using SYBR Green PCR Master Mix (Applied Biosystems) as described in the protocol. Small RNA and mRNA expression was normalized with 5S and GAPDH, respectively. Samples with a mean Ct>40 were assigned ‘Undetermined'. All qRT–PCR measurements were done on a 7900 HT instrument (Applied Biosystems). MAPK14 mRNA was detected using TaqMan Assay Hs01051152_m1 (Cat# 4331182 Applied Biosystems) and normalized to UBC.

### Cell viability and death assays

Cell viability was measured using the 3-[4,5-dimethylthiazol-2-yl]-2,5-diphenyltetrazolium bromide (MTT) assay (Roche Applied Science). Cellular death (LDH release) was measured using the Cytotoxicity Detection Kit PLUS (LDH) (Roche Applied Science). Fluorescence signal was measured using a multi-well ELISA reader (Synergy HT-reader, BioTek).

### Annexin V—PI apoptosis assay

For the apoptosis assay, cells were DOX-induced and treated with 64 μM oxPt for 48 h. Adherent and non-adherent cells were collected, pooled and stained using the Annexin V-FITC Apoptosis Detection Kit (Sigma-Aldrich) according to the manufacturer's protocol. Flow cytometry was performed at the FACS Core Facility, The Faculty of Health Sciences, Aarhus University, Denmark on a FACSAria IIII (BD Biosciences). FlowJo software version 8.8.3 (Tree Star Inc.) was used for data analysis. Initially, cells were gated with forward scatter-area (FSC-A) versus side scatter-area (SSC-A) followed by FSC-A versus forward scatter-height (FSC-H) to obtain cell singlets after which the percentage of cells in each quadrant of the fluorescein isothiocyanate (FITC) versus PI plot were obtained. For clarity only *n*=8,000 cells were visualized although typically at least 50,000 cells were counted.

### Anti-miR and siRNA experiments

For anti-miR experiments, cells were DOX-induced for 24 h prior transfection with anti-miR (MH12612, mirVana miRNA inhibitor (miRBase ID: hsa-miR-625-3p) catalogue (Cat.) #4464084, Life Technologies) or control miR (Pre-miR miRNA Precursor Molecules—Negative Control #2 Cat. #AM17111, Life Technologies) for 24 h before incubation in the presence of 0 or 64 μM oxPt for additional 48 h. To knock down MAPK14, we used SMARTpool, siGENOME MAPK14 siRNA (#M-003512-06-0005, Dharmacon Cat.). The cells were transfected with 20 nM siRNA 48 h prior LDH oxPt treatment. A scrambled siRNA (Cat. #4390843, Ambion) were transfected at 20 nM in parallel and used as control.

### AGO2 pull-down

The SW620.625 and control cells were scraped off culture flasks on ice in gentle lysis buffer (20 mM TRIS pH 7.5, 10 mM NaCl, 0.5% NP-40, 2 mM EDTA supplemented with RNase inhibitor RNaseOut (Life Technologies) and Complete Mini protease inhibitor cocktail (Roche Applied Science)), incubated for 5 min before being hypertonically lysed by increasing the NaCl concentration to 150 mM and incubated for additionally 5 min on ice. After 4 °C centrifugation at 19,000*g* for 10 min, the supernatant was collected and subjected to pull-down (10% was used for input control) by incubation with monoclonal AGO2 antibody 11A9 (Sigma-Aldrich, Cat. #SAB4200085)-bound Protein G-coupled Magnetic Dynabeads (Life Technologies; 15 mg 11A9 per 25 μl beads) following the manufacturer's recommendation. All washing steps were performed in ice cold washing buffer (50 mM TRIS pH 7.5, 150 mM NaCl and 0.05% NP-40), and total RNA from input and immunoprecipitate fractions purified with QIAzol (Qiagen). A parallel pull-down using monoclonal M2 anti-FLAG antibody (Sigma-Aldrich, Cat. #F1804) was performed as negative control.

### *miR-625-3p* luciferase reporter assay

Wild-type and mutated versions of part of the *MAP2K6* 3′UTR centred on the *miR-625-3p* target site were generated by primer extension PCR using oligos notI-map2k6.3UTR-rev and either of xhoI-map2k6.3UTR.wt-fwd, xhoI-map2k6.3UTR.mut1-fwd and xhoI-map2k6.3UTR.mut2-fwd (see Supplementary Data 3), and cloned into the psiCHECK-2 plasmid after the *Renilla* luciferase (*Rluc*) reporter gene. psiCHECK-2 contains Firefly luciferase, which enables accurate control of differences in transfection efficiency between experiments and wells. psiCHECK-2 vector without MAP2K6 3′UTR gave a robust signal and were used as mock control. 8000 HEK293T cells were transfected with Lipofectamin-2000 (Thermo Scientific) in 96-well plates using 50 ng mock, 3′UTR wild-type or mutated vectors alone, or cotransfected together with 20 nM *miR-625-3p* pre-miR (Life Technology, #AM17100) or scramble control pre-miR (Negative Control2 Life Technology #AM17111). One day after transfection, the cells were lysed and Renilla and Firefly substrates added using the Dual-Glo Luciferase Assay System (Promega #E2920) following the manufacturer's recommendations, and luminescence read in a multi-well ELISA reader (Synergy HT-reader, Bio-tek). *RLuc* signals were normalized to Firefly luminescence.

### Microarray profiling and RNA data processing

Expression profiling on cell lines was performed on three biological replicates on total RNA (all with RIN=10) isolated from SW620.625 and SW620.ctrl cells treated with 50 ng ml^−1^ DOX for 48 h using GeneChip Human Gene 2.0 ST arrays (Affymetrix) according to manufacturer's recommendation. Expression profiling on clinical samples was performed on RNA (median RIN=6.5) from pure cancer epithelium obtained using laser microdissection as described^52^. RNA was amplified using the Ovation Pico WTA system (Nugen) and profiled on GeneChip Human Gene 1.0 ST arrays (Affymetrix) according to manufacturer's recommendation. All samples passed basic quality control measures as performed in Affymetrix Gene Expressing Console. The data were loaded into the GeneSpring v.12.5 software (Agilent Technologies), and probe intensities quantile normalized and summarized into probe set values using the IterPLIER16 algorithm. Probe sets were excluded if they had (i) a mean log_2_(expression) <5 in both SW620.625 and control cells; (ii) an association with multiple gene symbols (that is, overlapping genes); (iii) no association with a gene symbol; (iv) an association with gene symbols mapping to several probes sets. Genes with a fold change >1.5 were considered dysregulated. Unsupervised hierarchical cluster analysis on SW620.625 and SW620.ctrl expression data were done in Cluster3 (ref. 56) using the most variable probe sets (that is, with a variance on the log_2_ expression values >0.25 across all the six samples) applying an absolute correlation similarity metric, and visualized using Treeview^57^. To identify putative *miR-625-3p* target genes, 3′UTR sequences were obtained from TargetScan (6.2) for all human annotated transcripts, and the longest 3′UTR sequence was chosen for isoforms with identical gene symbol. Genes were ranked by fold change in expression between *miR-625-3p* induction and scrambled control. Subsequently, 3′UTR sequences were scored for the presence of the target motif complementary to the seed sequence of *miR-625p-3p* (that is, CTATAGT). The empirical distribution of the ranks for genes with and without the target motif was compared using a Kolmogorov–Smirnov test. The web-based miRmap tool (http://mirmap.ezlab.org/app/)^8^ was used with standard parameters using the options ‘Species'=Human and ‘miRNA'=hsa-miR-625-3p; Candidate target genes with a miRmap score >75 were extracted.

### SILAC labelling and phosphopeptide enrichment

The HCT116.625 and HCT116.ctrl cells were grown in SILAC RPMI 1640 medium (PAA, Cat. #E15-087) with 2 mM L-glutamine and 10% dialysed fetal bovine serum (Sigma, Cat. #F0392) supplemented with heavy isotopes Arg10-13C6,15N4 (1.14 mM) and Lys8-13C6,15N2 (0.22 mM; Cambridge Isotope Laboratories, Cat. #CNLM-539-H and #CNLM-291-H), medium isotopes Arg6-13C and Lys4-D4 (Cat. #CLM-2265-H and #DLM-2640-O) or light isotopes Arg0 and Lys0 (Sigma). After >6 cell passages, we ensured that the incorporation rate was >95%. We also ensured that the HCT116.625 cells retained oxPt resistance after DOX induction compared with the HCT116.ctrl cells. The cell triplicates were labelled, induced with DOX and exposed to 16 μM oxPt according to the protocol described in Fig. 6a and Supplementary Fig. 10. Total protein lysates were harvested after several washes in ice-cold PBS using radioimmunoprecipitation assay buffer (50 mM TRIS pH 7.5, 150 mM NaCl, 50 mM EDTA, 0.1% sodium deoxycholate, 1% NP-40) supplemented with 1 mM Na-orthovanadate, 5 mM NaF, 5 mM β-glycerophosphate and complete Protease Inhibitor Cocktail (Roche). Lysates were pelleted at 4 °C at 15,000*g* for 15 min and the supernatant transferred to ice-cold acetone. The precipitated proteins were resuspended in 6 M urea, 2 M thiourea and 10 mM HEPES pH 8.0 and concentration was estimated with Bradford assay. Two triple SILAC experiments were designed to cover all conditions (Supplementary Fig. 10). After mixing proteins 1:1:1, these were reduced in 1 mM dithiothreitol followed by alkylation with 5 mM chloroacetamide, both steps for 45 min. The mixtures were pre-digested with LysC (Wako) in an enzyme/protein ratio of 1:100 (w/w) for 3 h followed by dilution with 50 mM ABC pH 8.0 to 2 M urea and further digested overnight with trypsin 1:100 (w/w). The digestion was quenched with trifluoroacetic acid TFA to a final concentration of 2% and the peptide mixture was washed and eluted from Sep-Pak (C18 Classic Cartridge, Waters). Elution was done with 2 ml 40% acetonitrile (ACN), 0.1% TFA followed by 4 ml 60% ACN, 0.1% TFA. The sample volume was doubled by addition of 12% TFA in ACN and subsequently enriched with TiO_2_ beads (5 μm, GL Sciences Inc., Tokyo, Japan) as previously described^58^, and finally enriched for a second and third time.

### MS/ms—proteome and phosphoproteome processing

The peptide mixture was separated on an in-house made 50 cm capillary column packed with 1.9 μm Reprosil-Pur C18 beads (Dr Maisch, Germany) using an EASY-nLC 1,000 system (Thermo Scientific). The column temperature was maintained at 50 °C using a column oven (PRSO-V1, Sonation GmbH, Biberach, Germany) and the LC system was interfaced online with the Q Exactive mass spectrometer (Thermo Scientific). Formic acid 0.1% was used to buffer the pH in the two running buffers used. The total gradient was 250 min followed by a 15 min washout and re-equilibration. In detail, the flow rate started at 250 nl min^−1^ and 5% ACN with a linear increase to 25% ACN over 220 min followed by 30 min linear increase to 60% ACN. The washout followed with 60% ACN for 5 min followed by re-equilibration with a 5 min linear gradient back down to 5% ACN, which were maintained for the last 5 min. For phosphopeptide-enriched samples, the Q Exactive was operated with a data-dependent method using Top10. Full scan resolutions were set to 70,000 at 200 *m*/*z* with a target value of 3 × 10^6^ and a maximum fill time of 20 ms. Mass range was set to 300–1,750 *m*/*z*. Fragment scan resolution were set to 35,000 with target value 1 × 10^5^ and maximum fill time 108 ms. Proteome data were acquired with a Top12 method and fragment scan resolution 17,500 and 44 ms fill time. Isolation width was 2 *m*/*z* and normalized collision energy (NCE) 28 for phosphor-enriched samples and 2.2 *m*/*z* and 25 NCE for proteome samples. All raw LC-MS/MS data were analysed by MaxQuant v1.4.1.4 (ref. 59), and searched against the human Uniprot database (April 2012 release). Carbamidomethylation of cysteine was specified as fixed modification for both groups. For the proteome data, variable modifications considered were oxidation of methionine, protein amino (N)-terminal acetylation and pyro-glutamate formation from glutamine. The phosphoproteome data were additionally searched with phosphorylation as a variable modification of serine, threonine and tyrosine residues. The match between run option was enabled, and the minimum score for both modified and unmodified peptides were set to 25, we used false discovery rate limit of 1% on peptide level.

### Proteome and phosphoproteome data analyses

From the two (HCT116.625 and HCT116.ctrl) triplicate proteome intensity data, we made log_2_(625/ctrl) ratios, and used only distinct proteins that were detected in all three ratios (*n*=2,410). All proteins with an absolute log_2_(625/ctrl) >0.58 are listed in Supplementary Data 2. SW620 proteome data were generated by separating protein lysates on a denaturing Bis-Tris gel, excise proteins between 20 and 45 kDa, which were then subjected to in-gel digestion followed by nLC-MS^60^. In HCT116 cells, MAP2K6 were quantified using peptides GAYGVVEK and INPELNQK shared with the MAP2K3 paralogue (since no MAP2K6 specific peptides were detected), whereas we used the MAP2K6 specific peptide DVKPSNVLINALGQVK in SW620 cells. Phosphopeptide positions were mapped to the Homo sapiens canonical UniProtKB data set^61^. We used HGNC gene names obtained from UniProtKB, which together with the phosphorylation positions acted as unique identifiers. Log_2_ ratios of normalized phosphopeptide intensities were generated for each triplicate ctrl+OX/ctrl, 625/ctrl, 625+OX/ctrl and 625+OX/ctrl+OX experiment, and the mean log_2_ ratio calculated for phosphopeptides detected in at least two of three replicates, while singly detected phospopeptides were discarded. We used a *t*-test to test the null hypothesis of no difference, that is, H0: log_2_ ratio=0, and estimated the local false discovery rate to obtain the chance of individual log_2_ ratios being false positives^62^. The Enrichr tool^63^ was used to obtain enrichment scores (*P*-values) to KEGG pathways and GO-terms, using HGNC names as input. Only the top five (lowest *P* values) for each category were reported. To detect changes in kinase activities, we applied the KSEA framework developed by Cassado *et al.*^19^ This is based on assigning individual phosphopeptides to one or more substrate groups according to the kinase(s) known or believed to catalyse the phosphorylation. To obtain such information, we merged and manually curated three collections of kinase phosphorylation mappings obtained from the Human Protein Reference Database^20^, PhosphoSitePlus^21^ and PhosphoELM^22^. From this collection, we extracted those phosphorylated substrates detected in our experiments, and used this to make 25 substrate groups with at least 10 substrate members. The fractional delta-count (fcount) was then calculated as the number of substrates with increased (log_2_ ratio >0, N_I_) minus the number of substrates with decreased (log_2_ ratio <0, N_D_) phosphorylation divided by the total number of substrates in the group, that is: fcount=(N_I_−N_D_)/(N_I_+N_D_). A Benjamin–Hochberg corrected *P*-value from a hypergeometric test was calculated to indicate the statistical significance of obtaining N_I_ (and N_D_). In addition to the fcount measure, we also calculated the mean log_2_ ratio () for all substrate log_2_ ratios within individual substrate groups, and tested whether this diverged from the population (experimental) mean (μ) using the z statistics (*z*=(−μ)/(*s*/N^0.5^), where *s* is the population standard deviation and *N* the number of substrates in the group). The *Z* values were converted into two-sided *P* values via the standard normal distribution (*P*=2*Φ(−abs(*z*))), which were corrected for multiple testing with the Benjamin–Hochberg procedure. To find altered phosphorylation motifs by KSEA for the ctrl+OX/ctrl and 625+OX/ctrl+OX data, we first extracted 15 amino acids-windows (7+1+7) around the central phosphorylated amino acid for all serine and threonine phosphopeptides (detected in two out of three experimental triplicates). Proteins with phosphorylations within seven amino acids from the N or carboxy (C) terminus were discarded. Phosphopeptides were then subjected to the motif-x algorithm^64^ using the following parameters: ‘occurrences'=5, ‘significance'=0.000001 and ‘background'=IPI Human Proteome. This resulted in 84 different motifs based on 7,850 phosphopeptides detected in the experiment. We further restricted KSEA to 51 motifs that were detected at least 50 times. To find kinases whose activities were associated with individual substrates and motifs, we obtained the netwoKIN data set of kinase substrate mappings^33^, but restricted us to entries with a high confidence (arbitrarily chosen as a networkin_score >3), corresponding to 36,972 out of the total 304,338 distinct kinase substrates in networKIN. To predict kinase activities associated with the differentially phosphorylated motifs (R-pS, pS-D and pS/pTP-R-K) we simplified the 15 amino acid motifs to 11 amino acids (5+1+5) motifs to directly use the 11 amino acids-phosphorylation windows provided for substrates in the networKIN data. Note that this resulted in the ‘…….SP….K'. motif was removed from the analysis since it is minimally described by a 6+1+6 format. We counted the enrichment score (ES) for each motif being associated with a given kinase as: ES_m,k_=(*n*_m,k_/*N*_m_)/(*N*_k_/*N*), where *n*_m,k_ is the number of times a kinase k is mapped to a motif m, *N*_m_ is the total number of motifs m, *N*_k_ is the total number kinases k and *N* is the total number of kinase motif mappings. We used a BH-corrected *P* value from a two-sided Fisher's exact test as significance for the association, and for each motif selected the top-10 kinases with the smallest *P* value and with at least 100 observations for a motif (*n*_m,k_≥100). Due to overlap between motifs, this resulted in 39 distinct kinases.

### Statistical analysis

If not mentioned otherwise, a two-sided Student's *t*-test was performed to evaluate statistical significance of differences in means, and the Fisher's exact test used to test independence of count data. Experiments were performed at least three times and *P* values ≤0.05 were considered significant (indicated with ‘*' in figures).

### Data availability

Proteome data are available via ProteomeXchange with identifier PXD002172. Clinical and cell line expression data can be obtained via GEO with identifiers GSE83129 and GSE83131, respectively. The authors declare that all other data supporting the findings of this study are available within the article and its Supplementary Information Files or from the corresponding author upon reasonable request.

## Additional information

**How to cite this article:** Rasmussen, M. H. *et al.* *miR-625-3p* regulates oxaliplatin resistance by targeting MAP2K6-p38 signalling in human colorectal adenocarcinoma cells. *Nat. Commun.* 7:12436 doi: 10.1038/ncomms12436 (2016).

## Supplementary Material

Supplementary Information Supplementary Figures 1-16, Supplementary Tables 1-2 and Supplementary References.

Supplementary Data 1 Up- and downregulated genes after miR-625-3p induction in SW620 cells

Supplementary Data 2 Proteins dysregulated after miR-625-3p induction in HCT116 cells

Supplementary Data 3 Oligos used
