# Supplementary material for: miR-625-3p regulates oxaliplatin resistance by targeting MAP2K6-p38 signalling in human colorectal adenocarcinoma cells
Source: Nat Commun. 2016 Aug 16;7:12436. doi: 10.1038/ncomms12436 (PMC4990699; doi:10.1038/ncomms12436)
Supplement: Supplementary Information — Supplementary Figures 1-16, Supplementary Tables 1-2 and Supplementary References. [file ncomms12436-s1.pdf]

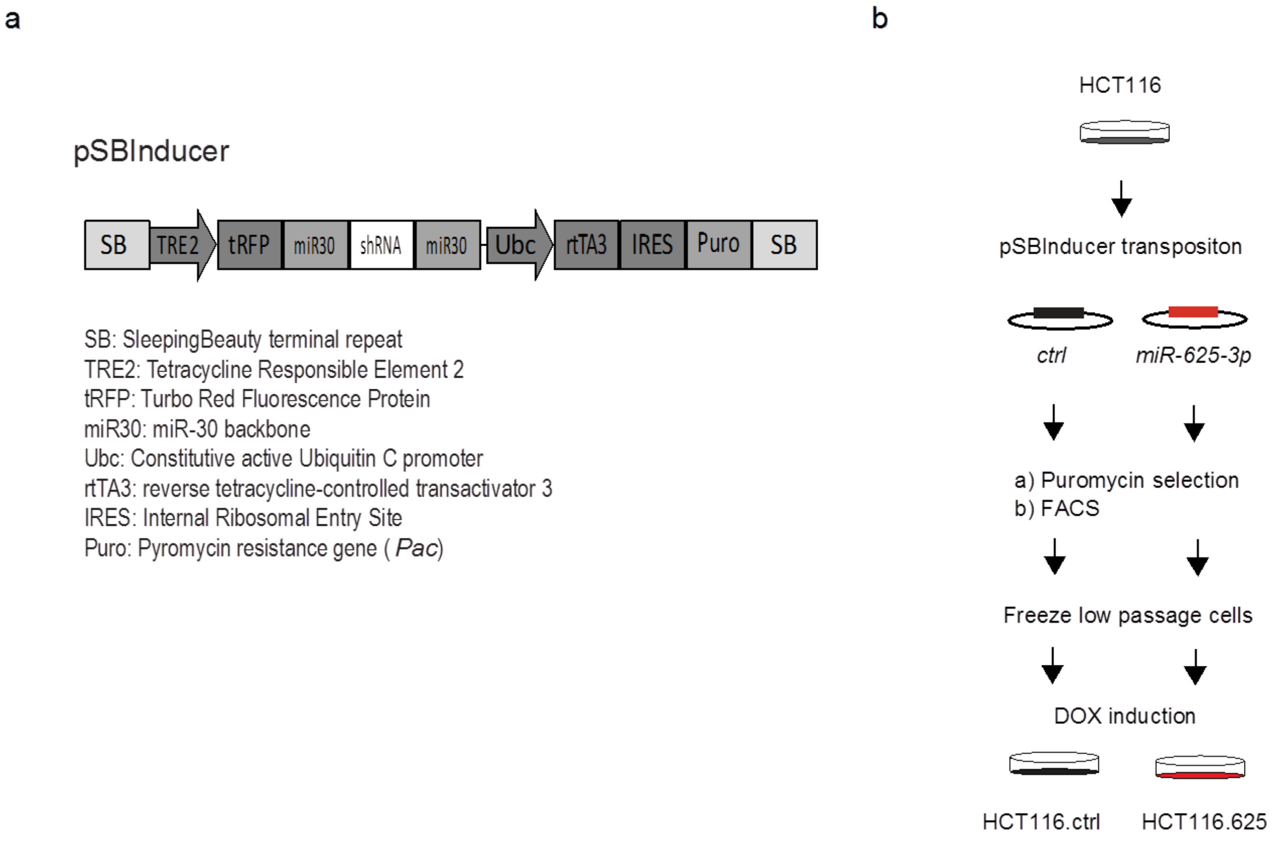

Supplementary Figure 1. **Diagram of pSBInducer vector and experimental design**

(a) The pSBInducer vector is based on the pINDUCER design [1], and enables transposase-mediated inducible expression of shRNAs. It consists of an expression cassette controlled by a doxycycline-inducible Tetracycline Responsible Element 2 (TRE2) minimal-promoter, which directs expression of the tRFP fluorescence reporter together with the short hairpin RNA (shRNA). The shRNA is embedded in the context of the well-characterized miR-30 structure, and expressed in the 3' untranslated region of tRFP. The constitutive active Ubc promoter drives the selection cassette containing reverse tetracycline-controlled transactivator 3 (rtTA3), an internal ribosomal entry site (IRES) and a puromycin selection marker or, alternatively, EGFP (not shown). SleepingBeauty (SB) inverted repeats flank the two cassettes to permit transposition into the host genome and thereby stable expression of the shRNA. Addition of doxycycline together with the constitutively expressed rtTA3 induces TRE2 leading to expression of tRFP and shRNA. (b) A schematic showing the generation of stable and DOX inducible shRNA/miRNA expressing cell lines as used throughout the paper, here exemplified with *miR-625-3p* expression in HCT116 CRC cells. First, pSBInducer plasmids encoding either a scrambled control shRNA or *miR-625-3p* are transfected into HCT116 cells together with a *SleepingBeauty* expressing plasmid (not shown) that catalyses random genomic integration of pSBInducer. Cells with stable integrations can either be obtained by puromycin selection or by fluorescence-activated cell sorting (FACS) depending on the selection marker (Puro in (a)). Using FACS single cell clones can also easily be obtained and expanded. After saving aliquots of low passage lines cells these can be used in down-stream analyses after induction of shRNA/miRNA expression with DOX.

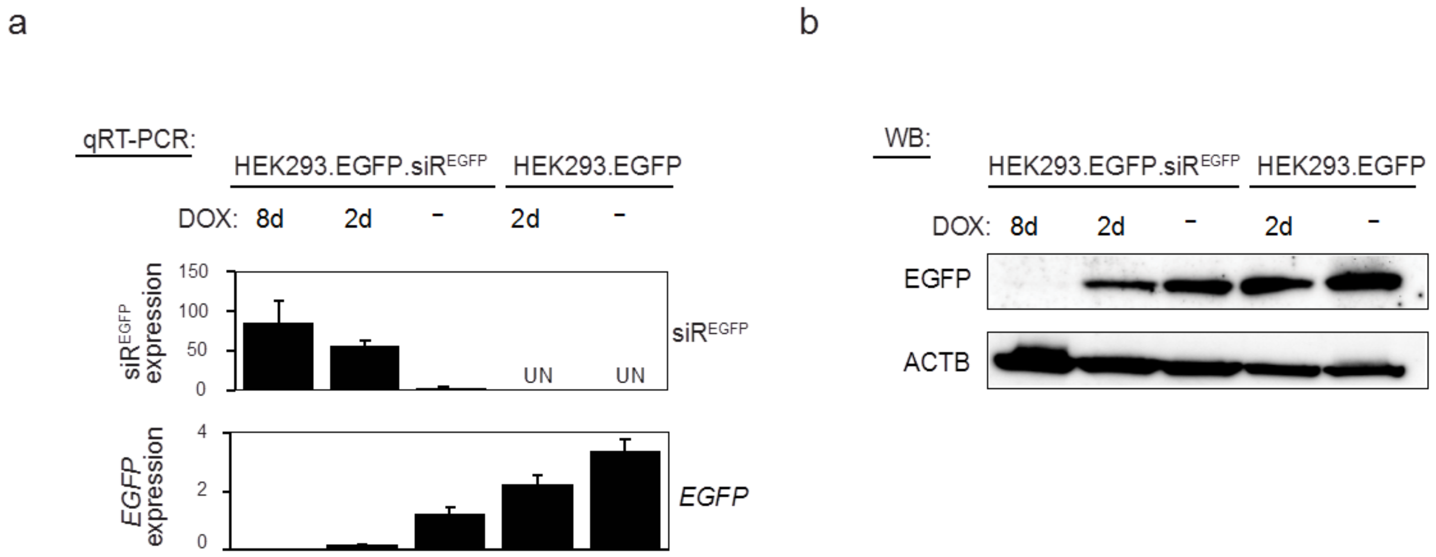

Supplementary Figure 2. Functional proof-of-concept of the pSBInducer vector

A pSBInducer vector expressing a shRNA against EGFP (siR^{EGFP}) was transposed into HEK293.EGFP cells that constitutively express EGFP [2]. After puromycin selection, HEK293.EGFP.siREGFP and HEK293.EGFP control cells were grown in normal (-) or inducible medium (50 ng/mL doxycycline) (DOX) for 2 and 8 days and total RNA and protein extracted. (a) Quantitative real time-PCR of siR^{EGFP} and EGFP (normalized to 5S and GAPDH, respectively). Displayed as mean ± SEM (n = 3). UN, 'Undetermined'. (b) Representative western blot analysis of EGFP protein levels on the same samples as in (a). Beta-actin was used as loading control.

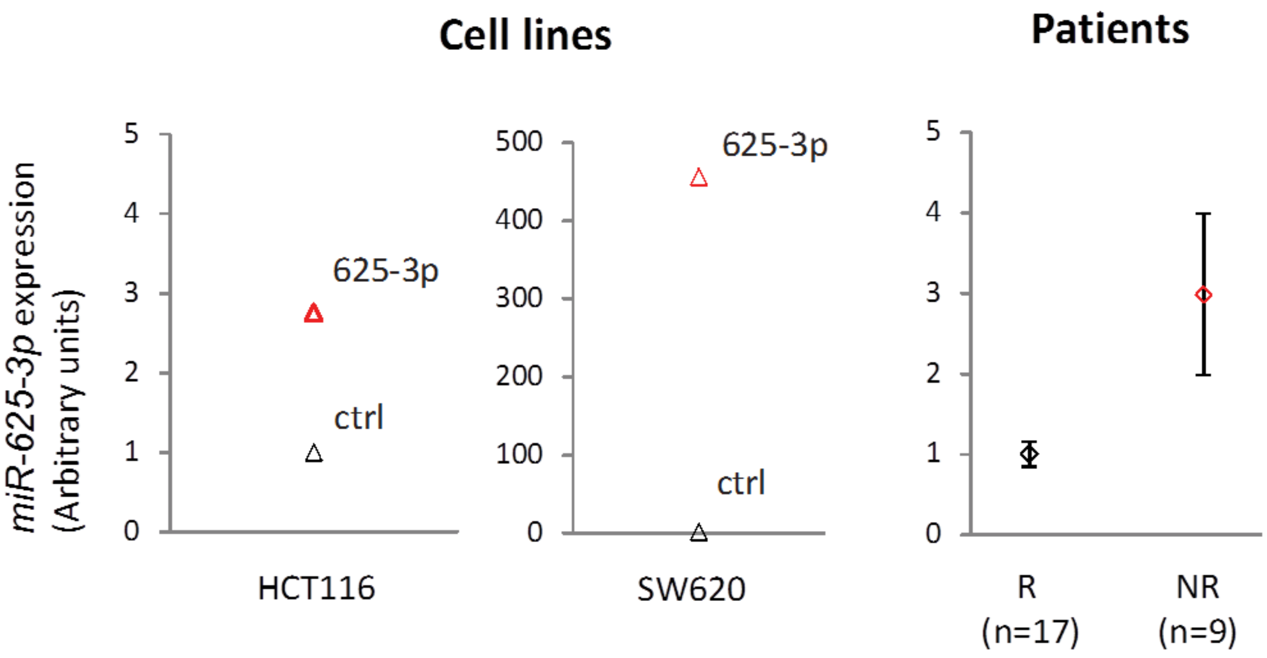

Supplementary Figure 3. *miR-625-3p* expression in pSBInducer CRC cells and oxPt treated patients
The levels of *miR-625-3p* was quantified in HCT116.625, SW620.625 and control cells by qRT-PCR after 48 hours of DOX induction, and compared to mCRC patients responding (R) and not responding (NR) to first-line oxPt based therapy. *miR-625-3p* data from patients was obtained from [3]. The difference in basal *miR-625-3p* levels in SW620.ctrl and HCT116.ctrl cells were small (the levels in HCT116.ctrl being ~30% lower than in SW620.ctrl), however to appreciate the difference between control and *miR-625-3p* expressing cells data is displayed as mean expression (arbitrary units) with control cells and responding patients set to 1. SEM of patient data is provided for the n=17 (R) and n=9 (NR) patients. Note the different scales on the *y-axes*.

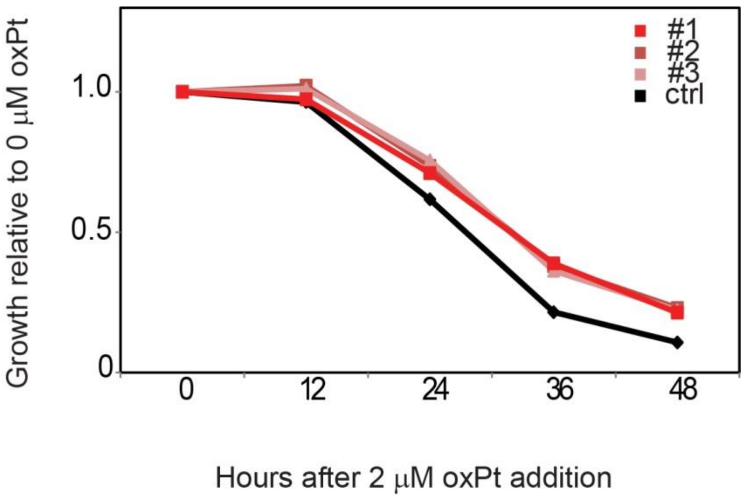

Supplementary Figure 4. *miR-625-3p* increases resistance to oxPt in single-cell derived HCT116.625 cells

Real time growth in 2 μM oxPt of individual single cell-derived HCT116.625 cells (clones #1-#3) was assessed using xCelligence real time assay. After 48 hours of DOX induction cells were transferred to xCelligence culture chambers where they were DOX induced for another day before oxPt containing medium were added. Real-time growth was measured for 48 hours, and every 12 hour data-point displayed (mean of n = 3 technical replicates).

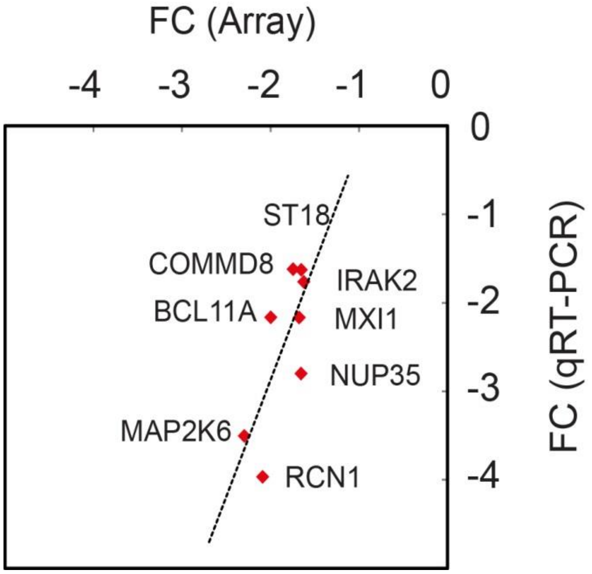

Supplementary Figure 5. Technical validation of *miR-625-3p* mRNA targets
As a technical validation of the down-regulation of candidate *miR-625-3p* target genes, a quantitative real-time PCR was done using the same RNA. The fold change (FC) as found for the array is displayed together with the FC found by qRT-PCR (normalized to *GAPDH*), and confirms the down-regulation of the eight candidate target genes.

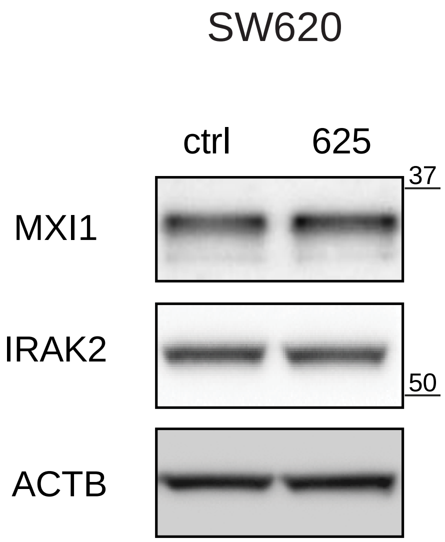

Supplementary Figure 6. No downregulation of MXI1 and IRAK2 after induction of *miR-625-3p*

Representative western blots of MXI1 and IRAK2 in SW620 cells after induction of *miR-625-3p* (or control) for 48 h. Beta-actin was used as loading control.

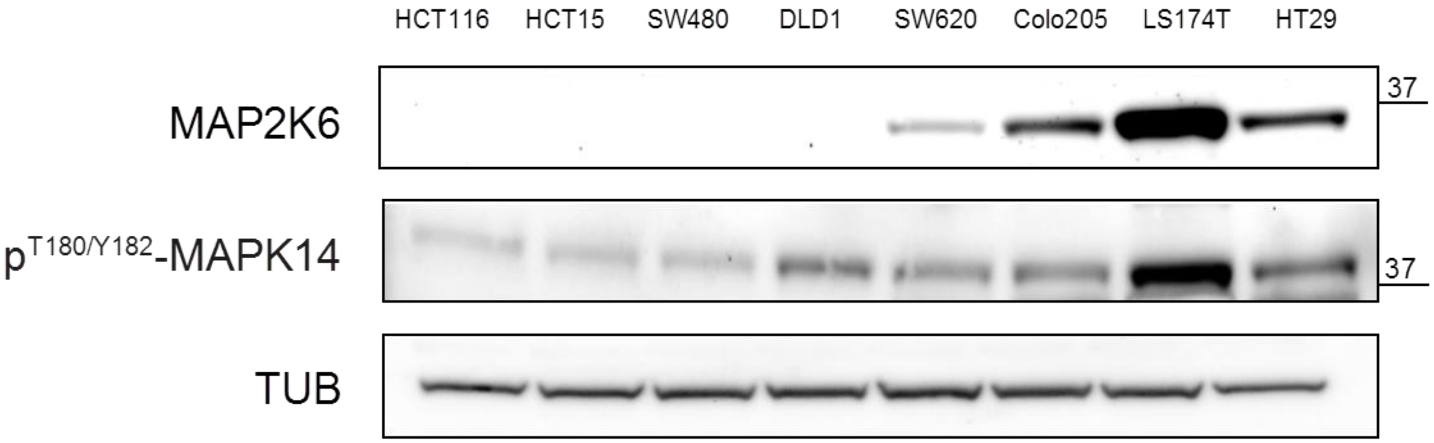

Supplementary Figure 7. Protein expression of MAP2K6 and p$^{T180/Y182}$-MAPK14 in eight different CRC cell lines

Representative western blots of MAP2K6 and p$^{T180/Y182}$-MAPK14 in eight different wildtype CRC cell lines with Tubulin as loading control. In HCT116 cells MAP2K6 is observable after prolonged exposure (see Fig 4b).

a

Number and type of detected proteins

Type	No
Kinase	177
Phosphatase	50
Other	2990
Total	*3217*

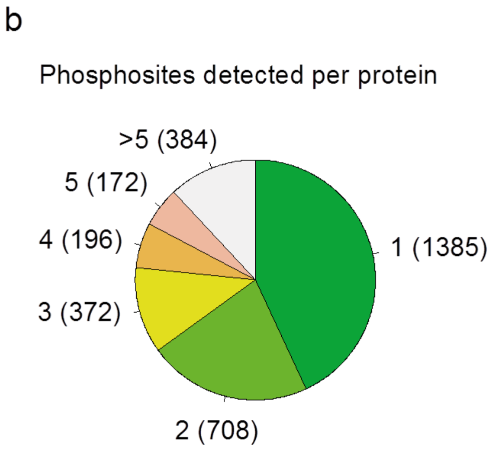

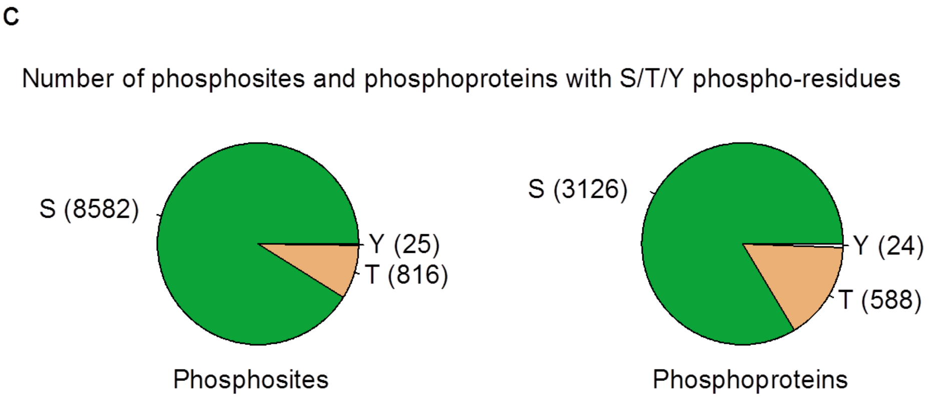

Supplementary Figure 8. SILAC phosphoproteome detection counts for proteins and phosphopeptides
(a) The number of distinct kinases, phosphatases and other phosphorylation substrates detected in the combined experiment ratios (625/ctrl, ctrl+OX/ctrl, 625+OX/ctrl and 625+OX/ctrl+OX). Information on known kinases was taken from KinBase (www.kinase.com) [4], and the known phosphatases from DEPOD (www.koehn.embl.de) [5]. (b) The number of proteins with the indicated number of different phosphorylation sites. (c) The distribution S, T and Y residue phosphorylations among the totally 9423 distinct phophopeptides detected (left). The number of S/T/Y residues per protein. Note that proteins may have a T or Y phosphorylation in addition to an S phosphorylation and hence the sum (3738) is larger than the total number of detected proteins (3217).

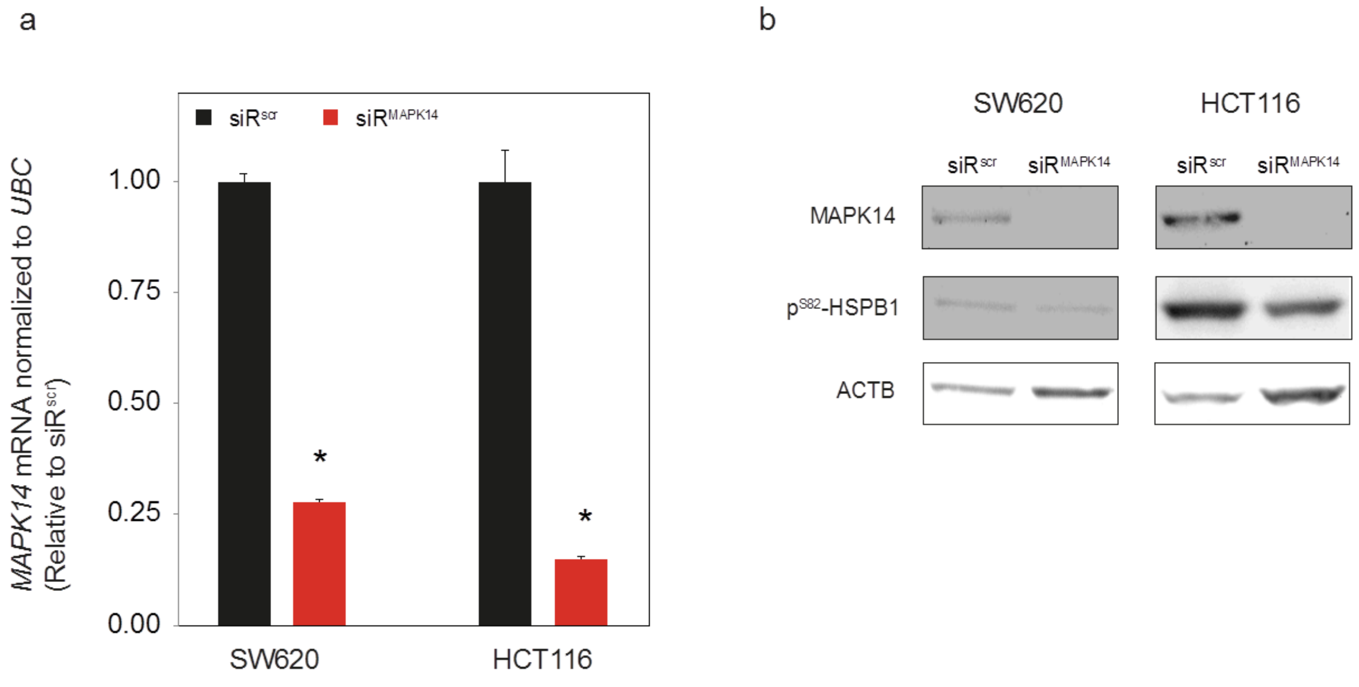

Supplementary Figure 9. siRNA mediated knock-down of MAPK14 results in decreased MAPK14 activity
(a) Transfecting SW620 and HCT116 cells with 20 nM of a pool of siRNAs targeting MAPK14 (siR^{MAPK14}) enabled reduction of *MAPK14* mRNA with 75 – 80 % as compared to cells transfected with scramble siRNA (siR^{scr}). Data was normalized to *UBC.* Displayed as mean *MAPK14* ± SEM from n = 3 technical replicates. (b) At the protein level MAPK14 was reduced to almost undetectable levels after 48 hours in SW620 cells and after 24 hours in HCT116 cells. In the same lysates phosphorylation of the MAPK14/MAPKAPK2 substrate $HSPB1^{Ser82}$ was clearly reduced as a consequence of MAPK14 removal supporting reduced MAPK14 signalling after knock-down. Beta-Actin was used as loading control.

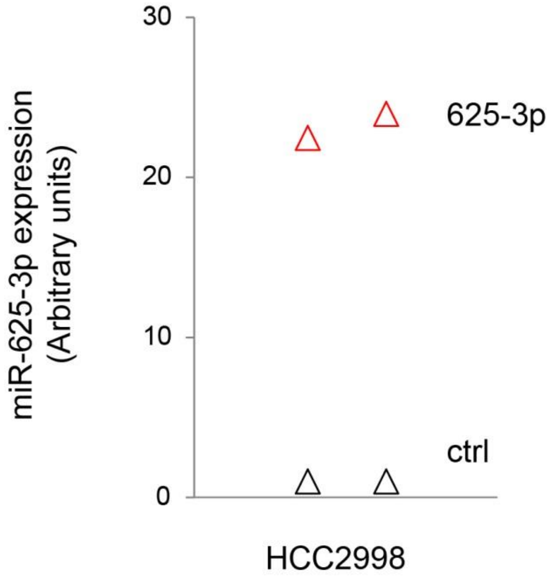

Supplementary Figure 10. ***miR-625-3p*** **expression in HCC2998.625 cells**
The levels of *miR-625-3p* were quantified in HCC2998.625 and control cells by qRT-PCR after 48 hours of DOX induction. Displayed are measurements from two technical replicates normalized to 5S (ctrl set to 1).

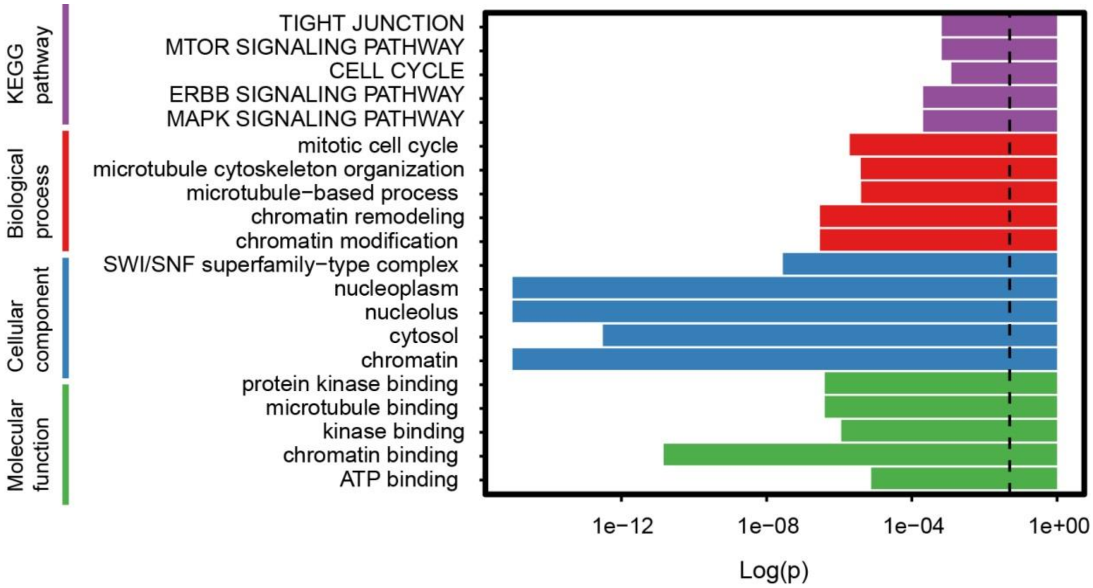

Supplementary Figure 11. Cellular processes associated with oxPt treatment of CRC cells

Dysregulated phosphoproteins involved in stress responses to 30 min of 16 μM oxPt treatment (as represented by phosphopeptides with an absolute fold change > 1.5 in the ctrl+OX/ctrl data set, and a FDR ≤ 0.1) were subjected to enrichment analysis to find significantly associated KEGG pathways as well as GO terms. Only the top-5 terms are displayed. Stippled line indicates adjusted p-value below 0.05.

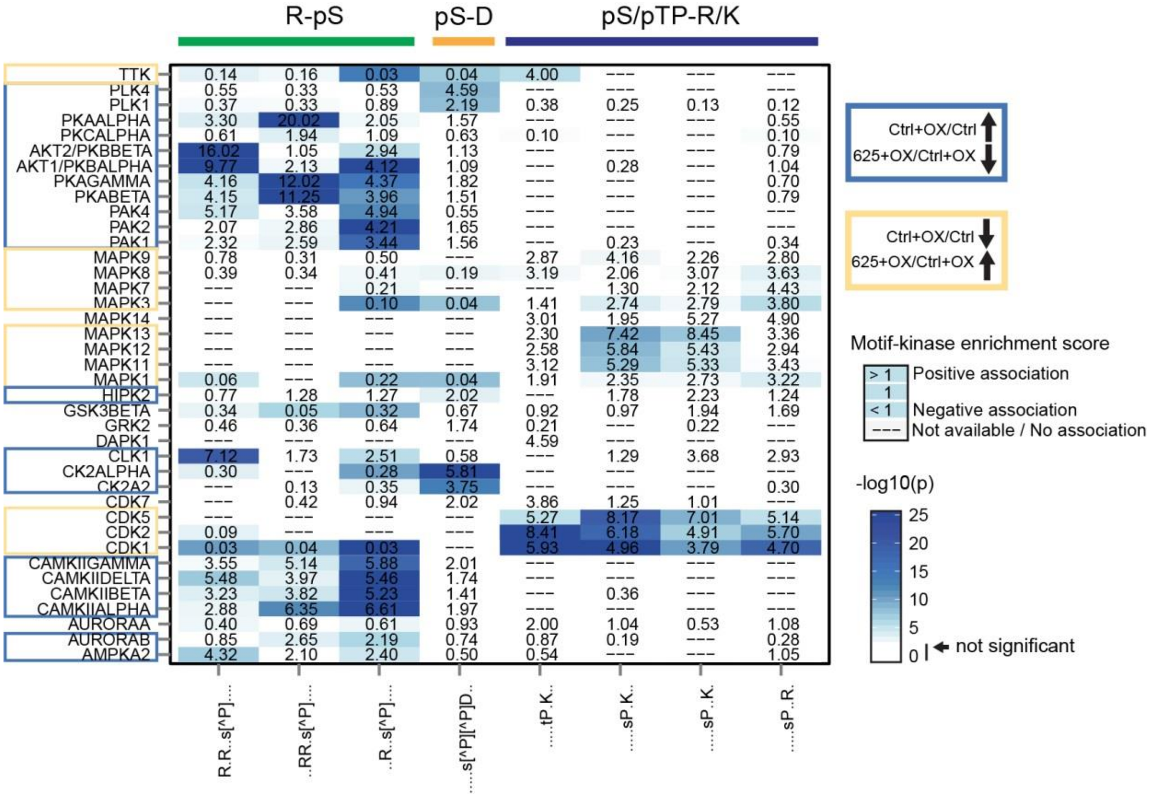

Supplementary Figure 12. Kinase-motif enrichment scores for OX- and *miR-625-3p* specific signalling

Enrichment scores (ES) for association between the differentially phosphorylation motifs and the networKIN kinase data set (see Methods). The motifs used to search the substrates of the networKIN data are shown below. The only constraint applied was not allowing a proline (indicated with [^P]) after the phosphorylation site in the R-pS and pS-D motifs. The degree of significance (Fisher's exact test, BH-corrected) is indicated by the colouring (ES with a $p > 0.05$ is not coloured). Enrichment scores for the 10 most significant kinases per motif (lowest p-value) associated with abundant numbers of predicted substrates (i.e. at least 100 predicted substrates in networKIN), yielded totally 39 distinct kinases. Kinases with increased predicted activity after oxPt treatment in HCT116.ctrl cells (ES > 1 and $p \leq 0.05$ for the R-pS or pS-D motifs) but decreased activity in oxPt treated HCT116.625 cells (ES < 1 and $p \leq 0.05$ for the pS/pTP-R/K motif) have been outlined in blue whereas kinases with a reverse pattern is outline in yellow.

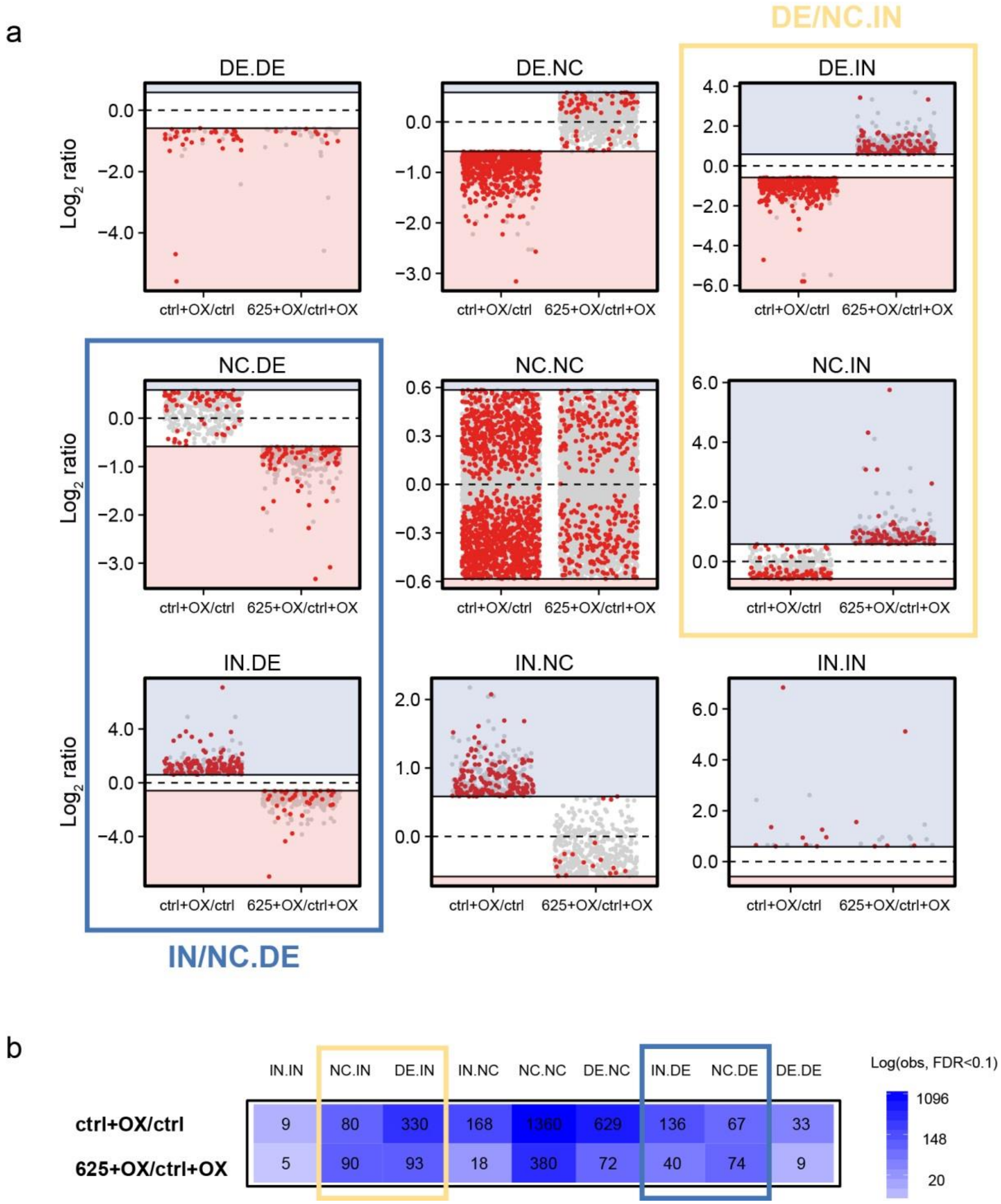
a
DE/NC.IN
DE.DE
DE.NC
DE.IN
NC.DE
NC.NC
NC.IN
IN.DE
IN.NC
IN.IN
IN/NC.DE
Log2 ratio
ctrl+OX/ctrl
625+OX/ctrl+OX
b
IN.IN NC.IN DE.IN IN.NC NC.NC DE.NC IN.DE NC.DE DE.DE
ctrl+OX/ctrl 9 80 330 168 1360 629 136 67 33
625+OX/ctrl+OX 5 90 93 18 380 72 40 74 9
Log(obs, FDR<0.1)
1096
148
20

Supplementary Figure 13. Phosphorylation classes after oxPt treatment

(a) Phosphopeptides detected in both the ctrl+OX/ctrl and 625+OX/ctrl+OX experiments were grouped into nine classes according to the combination of phosphorylation, i.e. whether phosphorylation was increased (IN, log2 ratio > 0.58, FDR ≤ 0.1), decreased (DE, log2 ratio < -0.58, FDR ≤ 0.1) or not changed (NC). Phosphopeptides with opposite phosphorylation patterns were defined as IN or NC in ctrl+OX/ctrl and DE in 625+OX/ctrl+OX (IN/NC.DE marked with blue), as well as DE or NC in ctrl+OX/ctrl and IN in 625+OX/ctrl+OX (DE/NC.IN marked with yellow). Phosphopeptides with FDR ≤ 0.1 are shown in red while phosphopeptides with FDR ≥ 0.1 are indicated in grey. (b) The number of significantly detected phosphopeptides (FDR ≤ 0.1) in the individual phosphorylation classes. Intriguingly, 39.8% of 781 625+OX/ctrl+OX phosphopeptides observed with a FDR ≤ 0.1 were differentially phosphorylated (IN or DE).

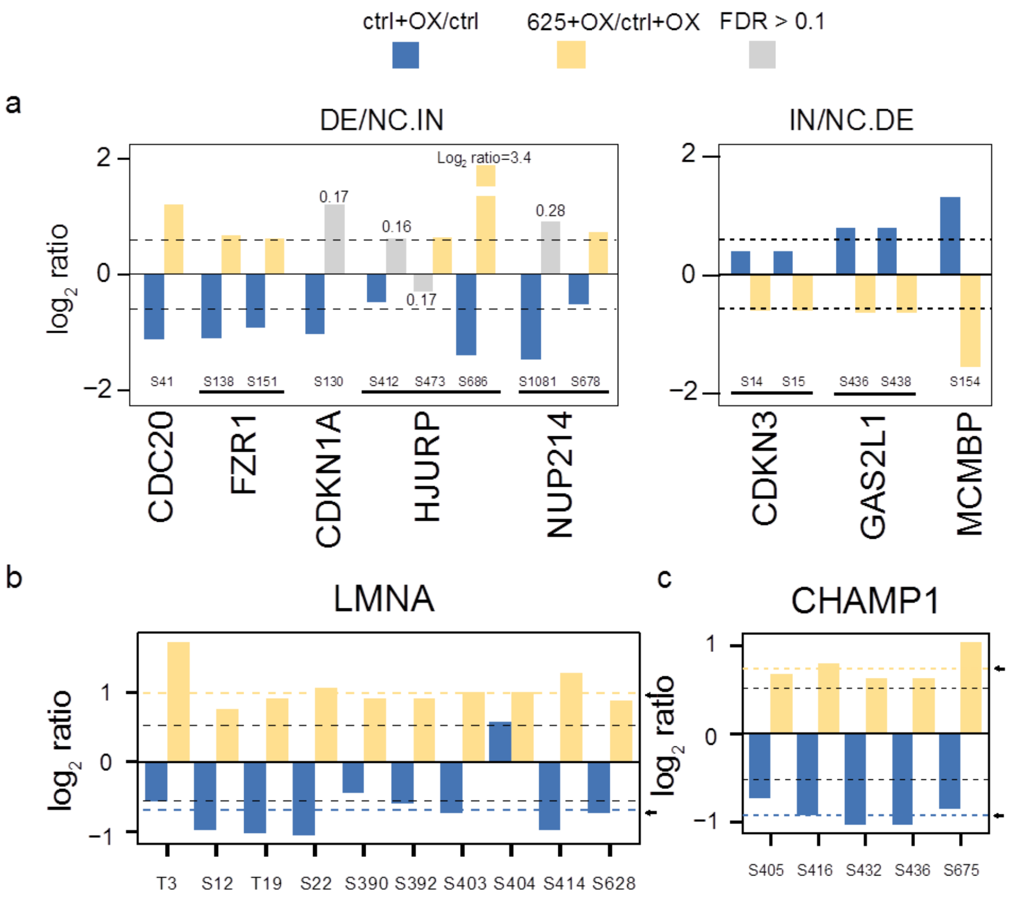

Supplementary Figure 14. Altered phosphorylation patterns in cells with increased *miR-625-3p* levels

(a) Phosphopeptides that showed a decrease (DE) or no change (NC) in phosphorylation in oxPt treated control cells (blue bars) but increased (IN) phosphorylation in oxPt treated HCT116.625 cells (yellow bars) (DE/NC.IN as defined in Supplementary Fig. 13) represented several essential cell cycle-regulatory phosphorylations. For instance, S41 phosphorylation of CDC20 [6] and S138/S151 phosphorylation of Fizzy-related protein homolog 1 (FZR1, also known as CDH1) [7, 8], the two key components determining ligase activity and specificity of anaphase-promoting complex/cyclosome APC/C [9]. We also found that HCT116.625 cells increased S130 phosphorylation of the cyclin-dependent kinase inhibitor CDKN1A ($p21^{Cip1}$), a phosphorylation mediated by CDK2/CCNE1 and which targets CDKN1A for ubiquitin ligase degradation [10]. In addition, oxPt treated HCT116.625 cells showed increased CDK1 mediated phosphorylation of the centrosome histone CENP-A charperone HJURP [11, 12], and increased phosphorylation of nuclear pore complex protein NUP214 [13]. These phosphorylations were decreased in HCT116.ctrl cells indicating less cell cycle progression. Phosphopeptides with the inverse regulation pattern (IN/NC.DE) included S13/S14 phosphorylation of CDKN3, which inhibits mitosis by dephosphorylating T162 on CDK2 [14], and S154 phosphorylation of MCMBP that are central for proper DNA replication [15]. Our data also suggest increased phosphorylation of S436 and S438 of growth arrest-specific 2 like 1 (GAS2L1) after oxPt treatment. For individual phosphopeptides a FDR > 0.1 is shown (grey bars). Note that the S686-HJURP bar has been truncated. (b) We found oxPt induced loss of phosphorylation throughout Lamin a/c (LMNA) in control HCT116 cells strongly indicative of decreased mitotic nuclear envelope disassembly [16, 17, 18]. In contrast, oxPt treated HCT116.625 cells displayed increased LMNA phosphorylation as compared to control cells. (c) A similar phosphorylation pattern was observed for Chromosome Alignment Maintaining Phosphoprotein 1 (CHAMP1), which recently was shown to be phosphorylated during mitosis in a CDK1-dependent manner [19]. In (b) and (c) we have only shown phosphopeptides with DE/NC.IN phosphorylation. Small arrows indicate the experimental mean log_2 ratios of the LMNA and CHAMP1 phosphorylations.

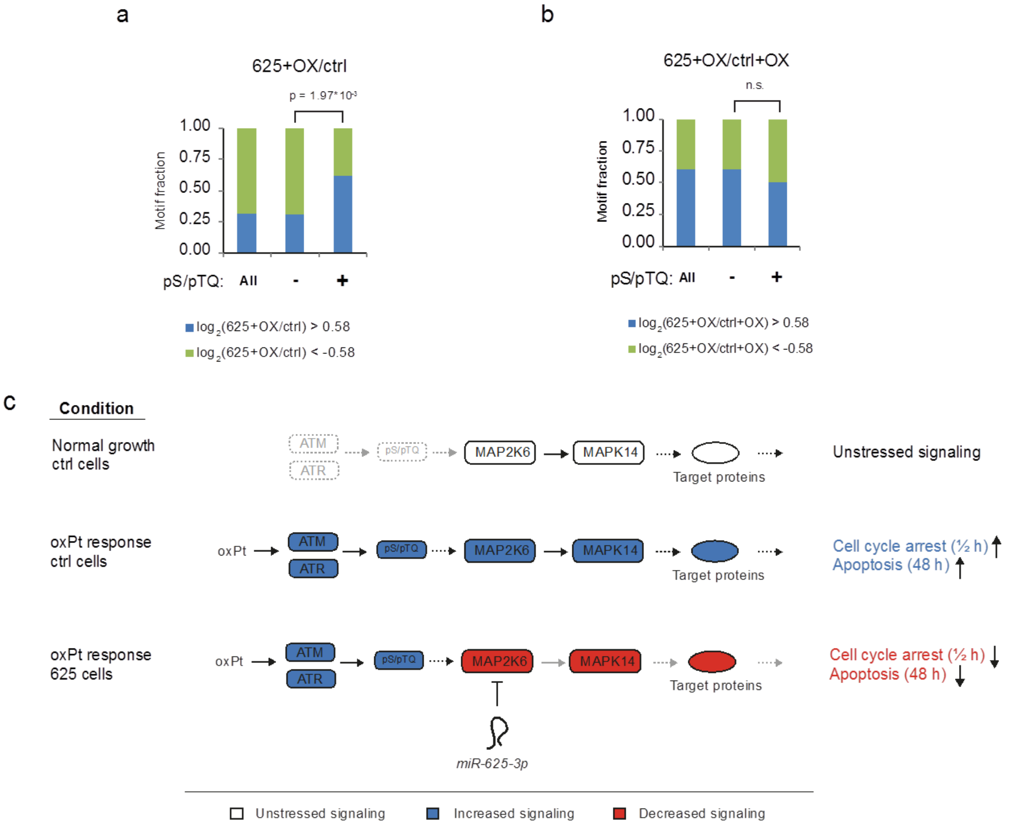

Supplementary Figure 15. Ectopic *miR-625-3p* expression does not compromise ATM/ATR signalling after oxPt treatment

(a) By looking at all significantly detected phosphopeptides with FDR ≤ 0.1, it was observed that oxPt treatment of HCT116.625 cells induced a general dephosphorylation ('All'), similar to what was seen in ctrl cells (Fig. 8c). In agreement, the fraction of phosphopeptides without a potential ATM/ATR DNA damage kinase motif (pS/pTQ) ('-') were predominantly dephosphorylated peptides (green). In contrast, among pS/pTQ motif containing phosphopeptides ('+'), a significant increased number of peptides became phosphorylated after oxPt treatment (blue) (Fisher's exact test). This indicated that increased *miR-625-3p* levels did not compromise the cell's ability to evoke DNA damage induced ATM/ATR signalling. (b) In the 625+OX/ctrl+OX experiment we found no difference in the distributions of pS/pTQ motifs among differentially phosphorylated peptides suggesting that the immediate ATM/ATR response after oxPt treatment is similar in HCT116.625 and control cells. (c) This indicates that the effect of ectopic *miR-625-3p* expression lies downstream of ATM/ATR signalling, in other words, that MAP2K6-p38/MAPK14 stress signalling is after ATM/ATR in CRC cells. Alternatively, it is possible that the *miR-625-3p*-MAP2K6-p38/MAPK14 resistance is independent of ATM/ATR signals (not drawn).

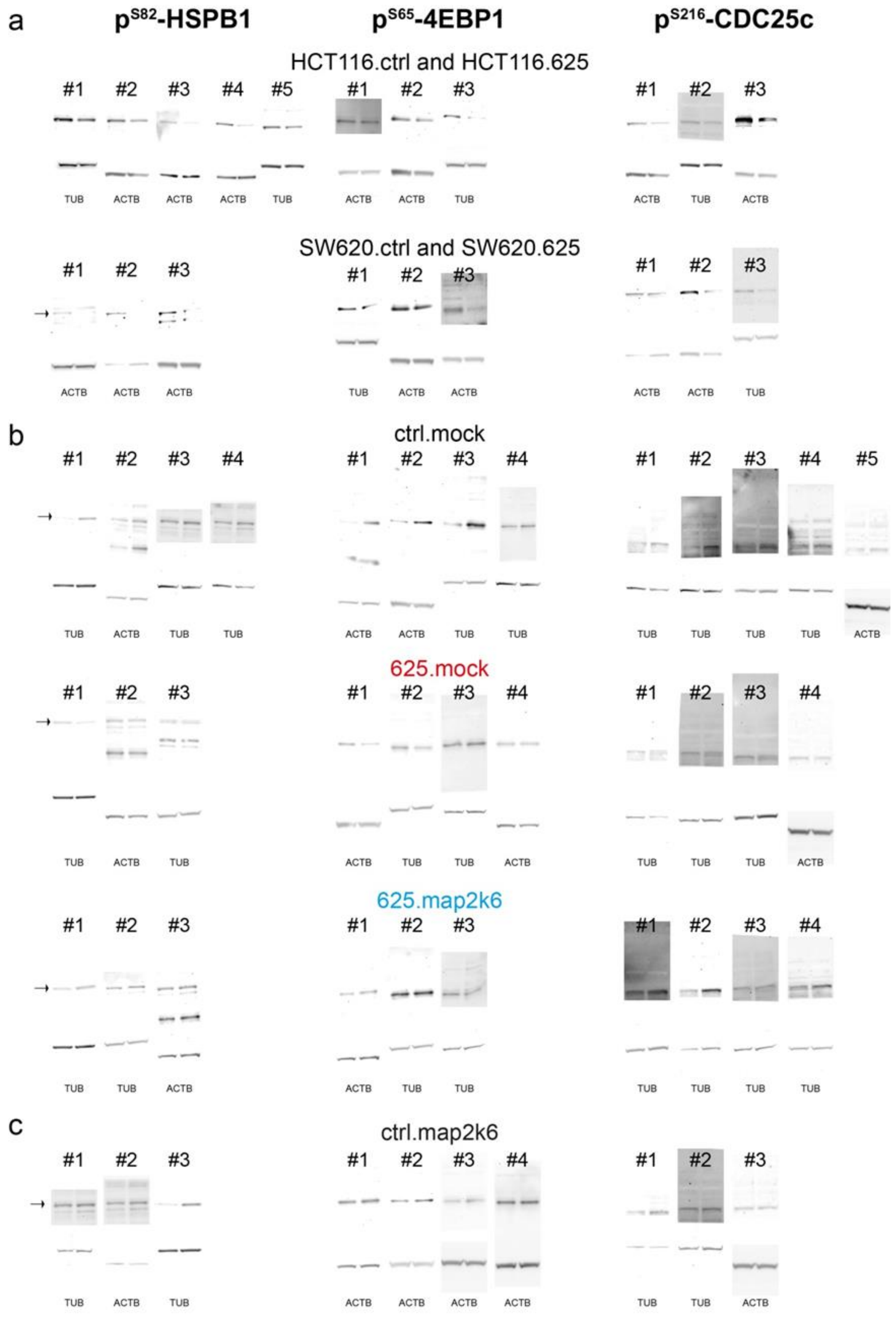

Supplementary Figure 16. Westerns blot used for densitometrical analysis. Unadjusted replicated scans as used in the semi-quantitative analyses of Fig. 5a (a), Fig. 5b (b) and Fig.5c (c). Samples as displayed in Fig. 5a-c.

Single cell clone	OxPt (64 μM)	Early apoptosis (Q1)	Late apoptosis (Q2)	Dead/Necrotic (Q3)	Live (Q4)	Death rate [a]
**ctrl**	-	2.0	2.6	2.6	92.9	
	+	2.9	19.4	32.4	45.3	51.2
miR-625-3p	-	0.2	2.9	2.7	94.3	
	+	9.9	12.3	14.4	63.4	32.8

a, Death rate = 100% * [1 – ($Q4_{64\ \mu M}$ / $Q4_{0\ \mu M}$)]

Supplementary Table 1. Annexin V-PI analyses of apoptosis in SW620 ctrl and *miR-625-3p* single cell-clones

We note that the decreased oxPt induced apoptosis in SW620.625 cells as detected by the Annexin V-PI assay appear discordant with the LDH assay observations (Fig. 2a). We believe it likely that specific quantification of extra-cellular phosphatidylserine exposure (as done in the Annexin-V/PI assay) is a more reliable and sensitive measure of oxPt cytotoxicity in SW620 cells than LDH release.

Affymetrix probe cluster	Gene	miRmap score [a]
16837348	MAP2K6	80.9
16723246	RCN1	84.5
16897946	BCL11A	78.5
16975659	COMMD8	83.2
16709108	MXI1	89.6
16888412	NUP35	82.2
17077073	ST18	92.8
16937579	IRAK2	90.2

Supplementary Table 2. Genes selected as putative *miR-625-3p* targets
The miRmap tool [20] was run using default parameters. The miRmap score represents a composite score of individual site's scores. For instance, the MAP2K6 3'UTR contains both a distal low-scoring (miRmap score = 49.14) and a proximal high-scoring (miRmap score = 85.49) *miR-625*-3p site.

Supplementary References

1. Meerbrey KL*, et al.* The pINDUCER lentiviral toolkit for inducible RNA interference in vitro and in vivo. *Proc Natl Acad Sci U S A* **108**, 3665-3670 (2011).

2. Moldt B, Staunstrup NH, Jakobsen M, Yanez-Munoz RJ, Mikkelsen JG. Genomic insertion of lentiviral DNA circles directed by the yeast Flp recombinase. *BMC biotechnology* **8**, 60 (2008).

3. Rasmussen MH*, et al.* High expression of microRNA-625-3p is associated with poor response to first-line oxaliplatin based treatment of metastatic colorectal cancer. *Mol Oncol* **7**, 637-646 (2013).

4. Manning G, Whyte DB, Martinez R, Hunter T, Sudarsanam S. The protein kinase complement of the human genome. *Science* **298**, 1912-1934 (2002).

5. Duan G, Li X, Kohn M. The human DEPhOsphorylation database DEPOD: a 2015 update. *Nucleic acids research* **43**, D531-535 (2015).

6. Tang Z, Shu H, Oncel D, Chen S, Yu H. Phosphorylation of Cdc20 by Bub1 provides a catalytic mechanism for APC/C inhibition by the spindle checkpoint. *Molecular cell* **16**, 387-397 (2004).

7. Fukushima H*, et al.* SCF-mediated Cdh1 degradation defines a negative feedback system that coordinates cell-cycle progression. *Cell reports* **4**, 803-816 (2013).

8. Qiao X, Zhang L, Gamper AM, Fujita T, Wan Y. APC/C-Cdh1: from cell cycle to cellular differentiation and genomic integrity. *Cell cycle* **9**, 3904-3912 (2010).

9. Yu H. Cdc20: a WD40 activator for a cell cycle degradation machine. *Molecular cell* **27**, 3-16 (2007).

10. Bornstein G, Bloom J, Sitry-Shevah D, Nakayama K, Pagano M, Hershko A. Role of the SCFSkp2 ubiquitin ligase in the degradation of p21Cip1 in S phase. *The Journal of biological chemistry* **278**, 25752-25757 (2003).

11. Dunleavy EM*, et al.* HJURP is a cell-cycle-dependent maintenance and deposition factor of CENP-A at centromeres. *Cell* **137**, 485-497 (2009).

12. Muller S, Montes de Oca R, Lacoste N, Dingli F, Loew D, Almouzni G. Phosphorylation and DNA binding of HJURP determine its centromeric recruitment and function in CenH3(CENP-A) loading. *Cell reports* **8**, 190-203 (2014).

13. Favreau C, Worman HJ, Wozniak RW, Frappier T, Courvalin JC. Cell cycle-dependent phosphorylation of nucleoporins and nuclear pore membrane protein Gp210. *Biochemistry* **35**, 8035-8044 (1996).

14. Poon RY, Hunter T. Dephosphorylation of Cdk2 Thr160 by the cyclin-dependent kinase-interacting phosphatase KAP in the absence of cyclin. *Science* **270**, 90-93 (1995).

15. Sakwe AM, Nguyen T, Athanasopoulos V, Shire K, Frappier L. Identification and characterization of a novel component of the human minichromosome maintenance complex. *Molecular and cellular biology* **27**, 3044-3055 (2007).

16. Heald R, McKeon F. Mutations of phosphorylation sites in lamin A that prevent nuclear lamina disassembly in mitosis. *Cell* **61**, 579-589 (1990).

17. Peter M, Nakagawa J, Doree M, Labbe JC, Nigg EA. In vitro disassembly of the nuclear lamina and M phase-specific phosphorylation of lamins by cdc2 kinase. *Cell* **61**, 591-602 (1990).

18. Ward GE, Kirschner MW. Identification of cell cycle-regulated phosphorylation sites on nuclear lamin C. *Cell* **61**, 561-577 (1990).

19. Itoh G*, et al.* CAMP (C13orf8, ZNF828) is a novel regulator of kinetochore-microtubule attachment. *The EMBO journal* **30**, 130-144 (2011).

20. Vejnar CE, Zdobnov EM. MiRmap: comprehensive prediction of microRNA target repression strength. *Nucleic acids research* **40**, 11673-11683 (2012).
